# Supplementary material for: Analysis of diverse eukaryotes suggests the existence of an ancestral mitochondrial apparatus derived from the bacterial type II secretion system
Source: Nat Commun. 2021 May 19;12:2947. doi: 10.1038/s41467-021-23046-7 (PMC8134430; doi:10.1038/s41467-021-23046-7)
Supplement: Supplementary file 1 — Supplementary Information [file 41467_2021_23046_MOESM1_ESM.pdf]

SUPPLEMENTARY FIGURES & METHODS

Ancestral mitochondrial apparatus derived from the bacterial type II secretion system

Horváthová et al.,

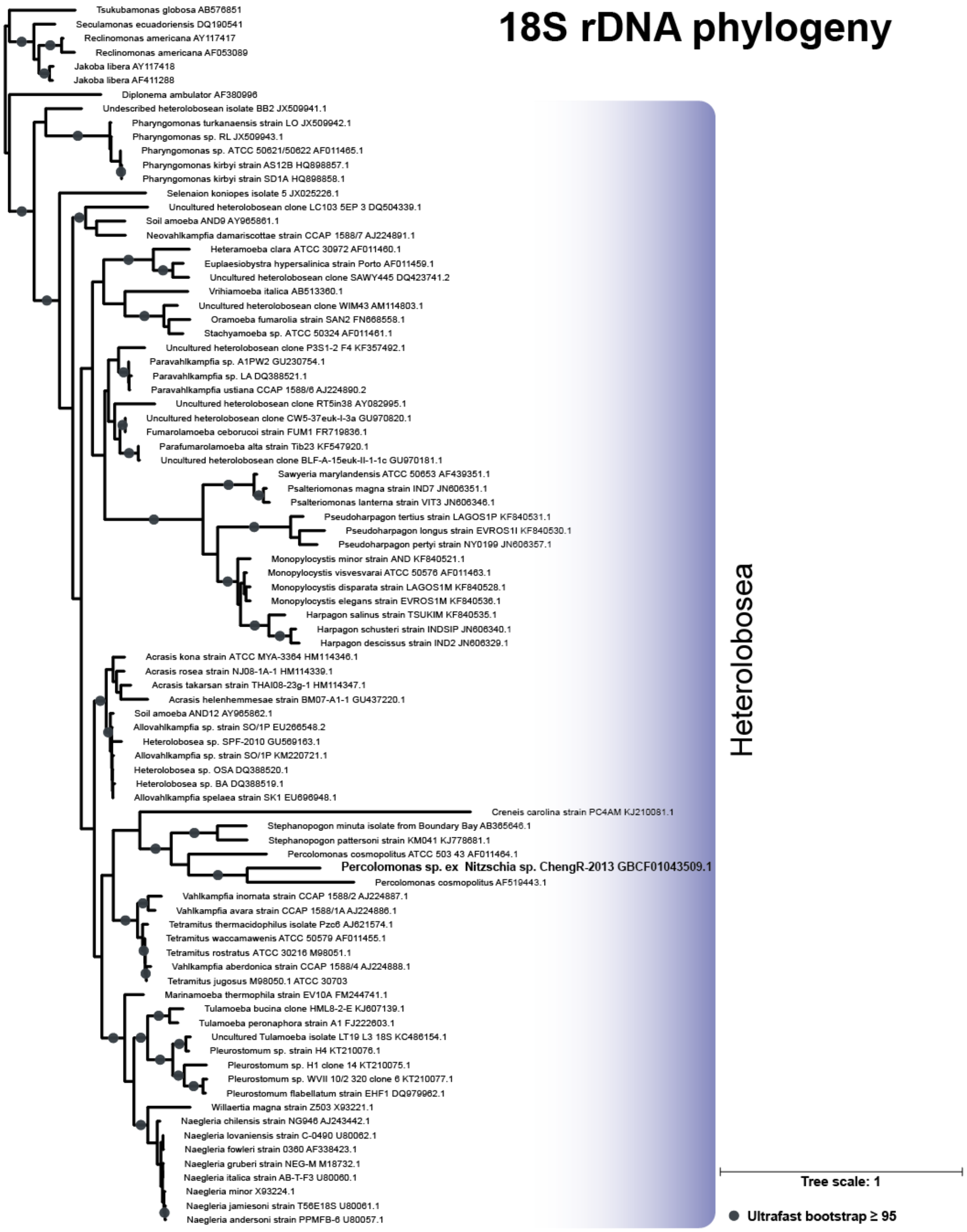

Supplementary Fig. 1 Phylogenetic analysis of 18S rRNA gene from Heterolobosea showing the position of three *Percolomonas* species for which transcriptome data are available. The ML tree was calculated using IQ-TREE (substitution model TIM2+I+G4) with 1000 ultrafast bootstraps. The two taxa currently identified as two strains (AE-1 and WS) of the same species of *Percolomonas cosmopolitus* and used for generating transcriptome assemblies in the context of the MMETSP project (MMETSP0758 and MMETSP0759, respectively; https://www.imicrobe.us/#/projects/104) are in fact very different at the level of the 18S rRNA gene sequence and apparently represent two different species. In addition, we found out that the transcriptome assembly of the diatom *Nitzschia* sp. ChengR-2013 (NCBI BioProject PRJNA243394) is massively contaminated by sequences from a heterolobosean that according to the phylogenetic analysis of the 18S rRNA sequence present in the assembly is specifically related to *P.*

cosmopolitus strain WS, yet again sufficiently different at the sequence level to be considered a separate species (provisionally denoted as *Percolomonas* sp. ex *Nitzschia* sp. ChengR-2013).

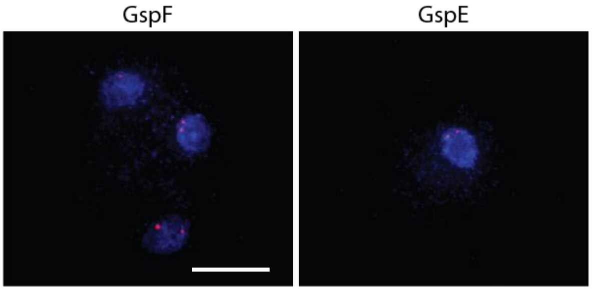

Supplementary Fig. 2 Fluorescence *in situ* hybridization (FISH) of selected Gsp genes in *N. gruberi* nuclei. Probes specific for the *Ng*GspE and *Ng*GspF genes each label two loci (red) in diploid nuclei (blue) of *N. gruberi*. DNA was stained with DAPI, blue dots correspond to mitochondrial DNA. (representative images of multiple experiments are shown). Scale bar 10 μm.

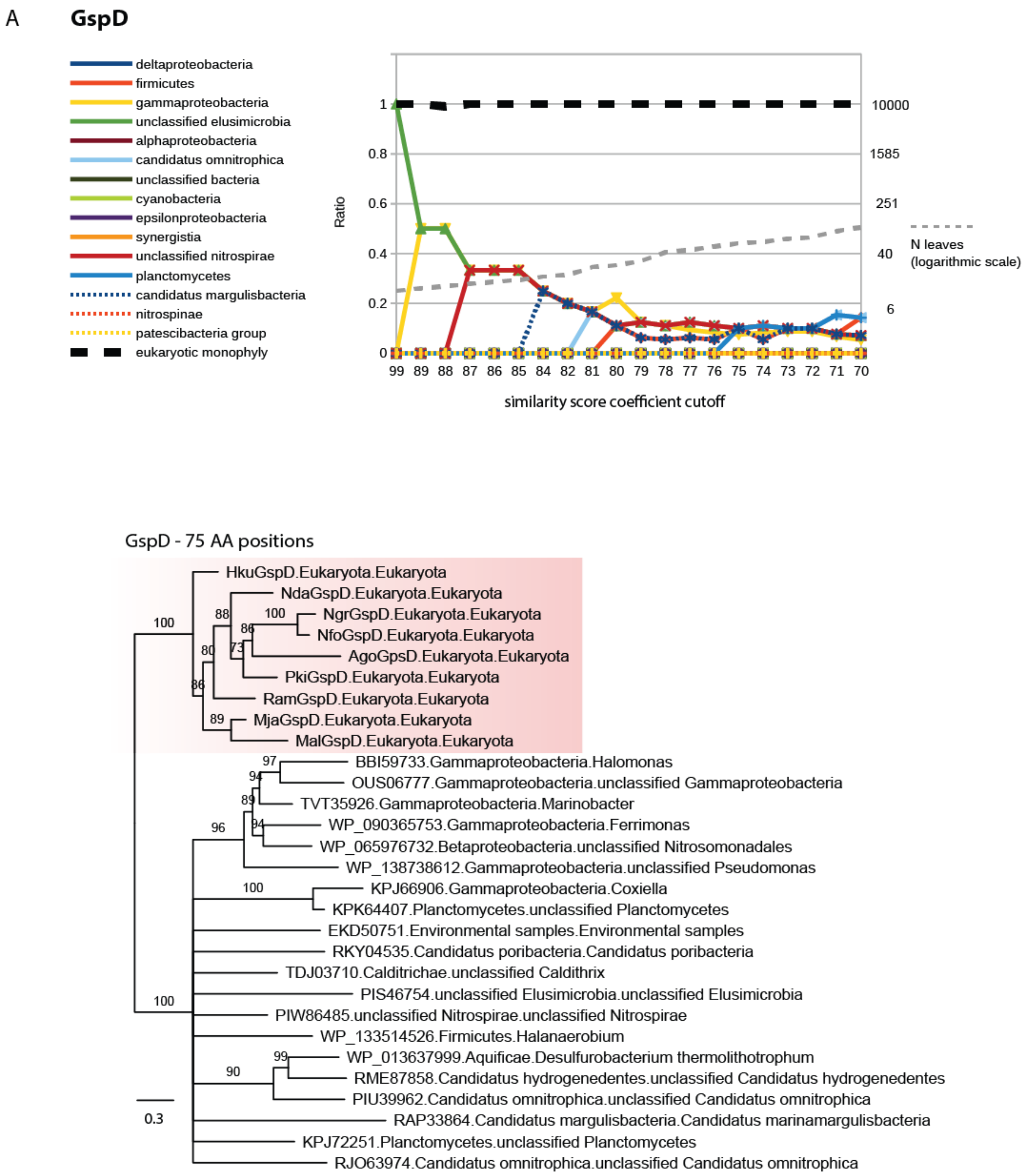

Supplementary Fig. 3A Phylogenetic relationship of the eukaryotic GspD proteins to prokaryotic homologues. For further explanations see page 7.

B **GspE**

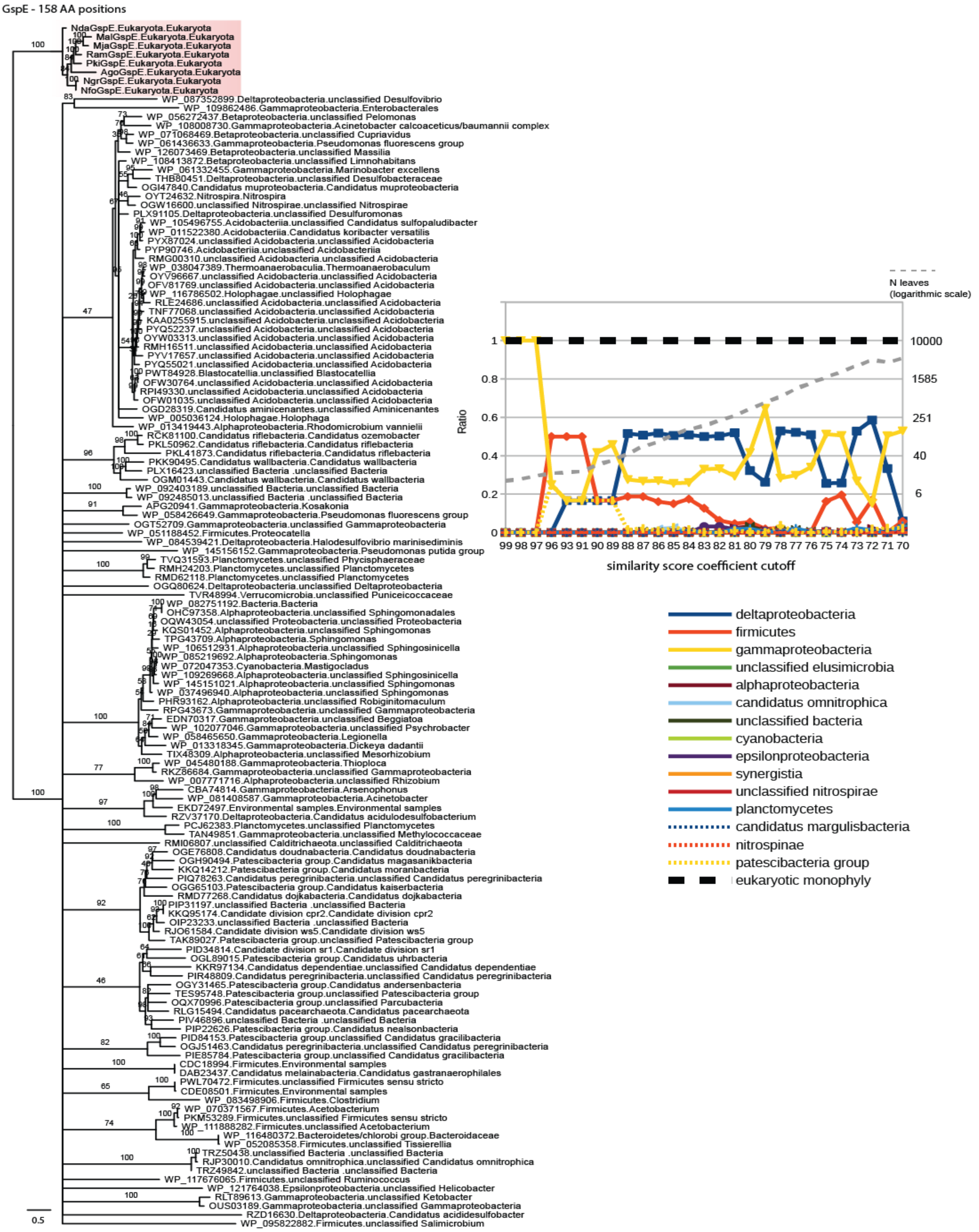

Supplementary Fig. 3B Phylogenetic relationship of the eukaryotic GspE proteins to prokaryotic homologues. For further explanations see page 7.

C **GspF**

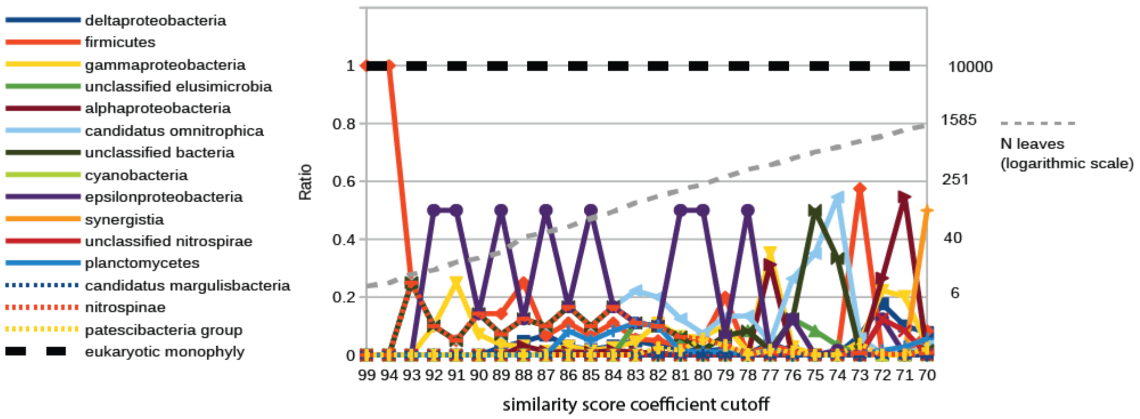

GspF - 287 AA positions

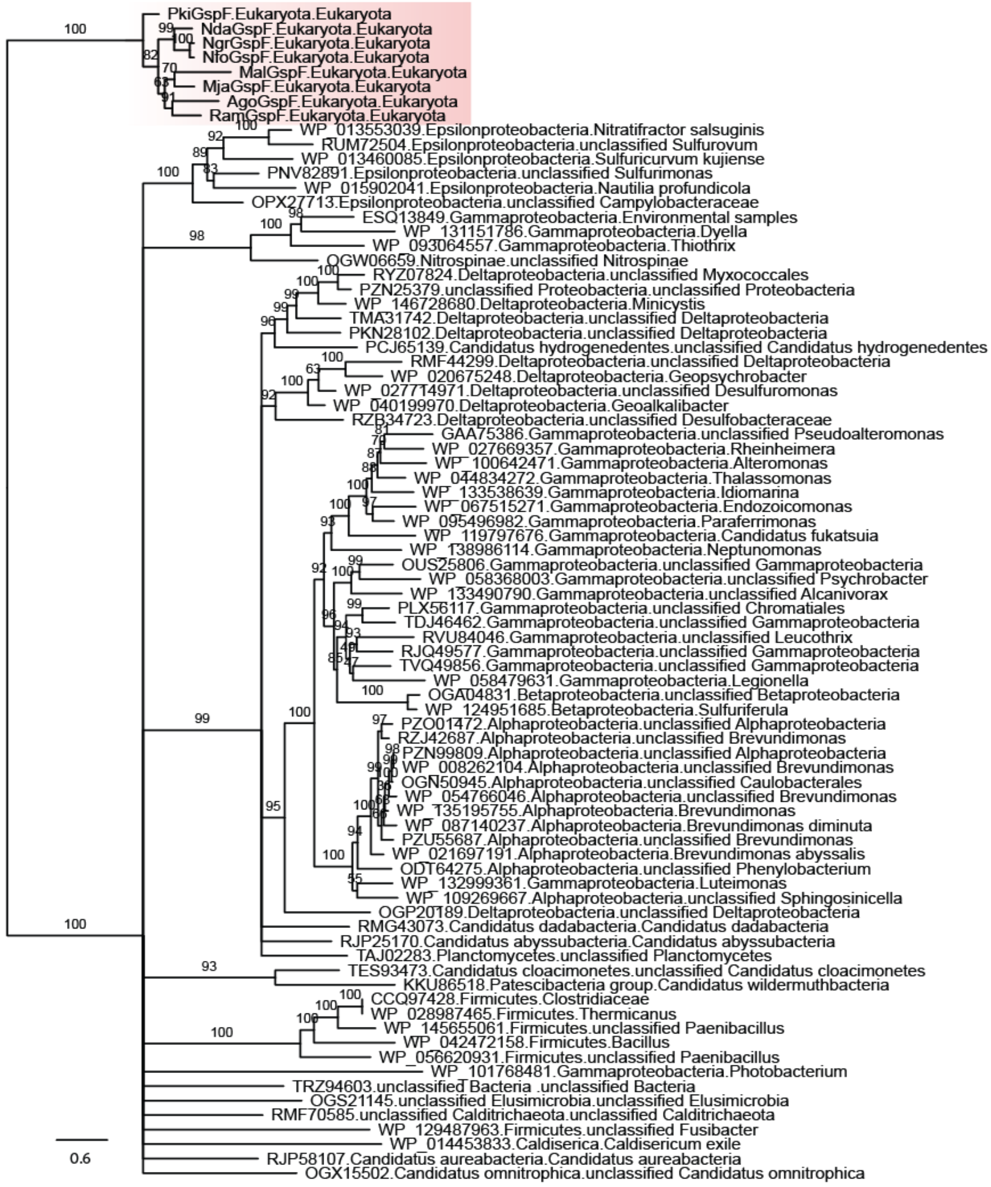

Supplementary Fig. 3C Phylogenetic relationship of the eukaryotic GspE proteins to prokaryotic homologues. For further explanations see page 7.

D **GspG**

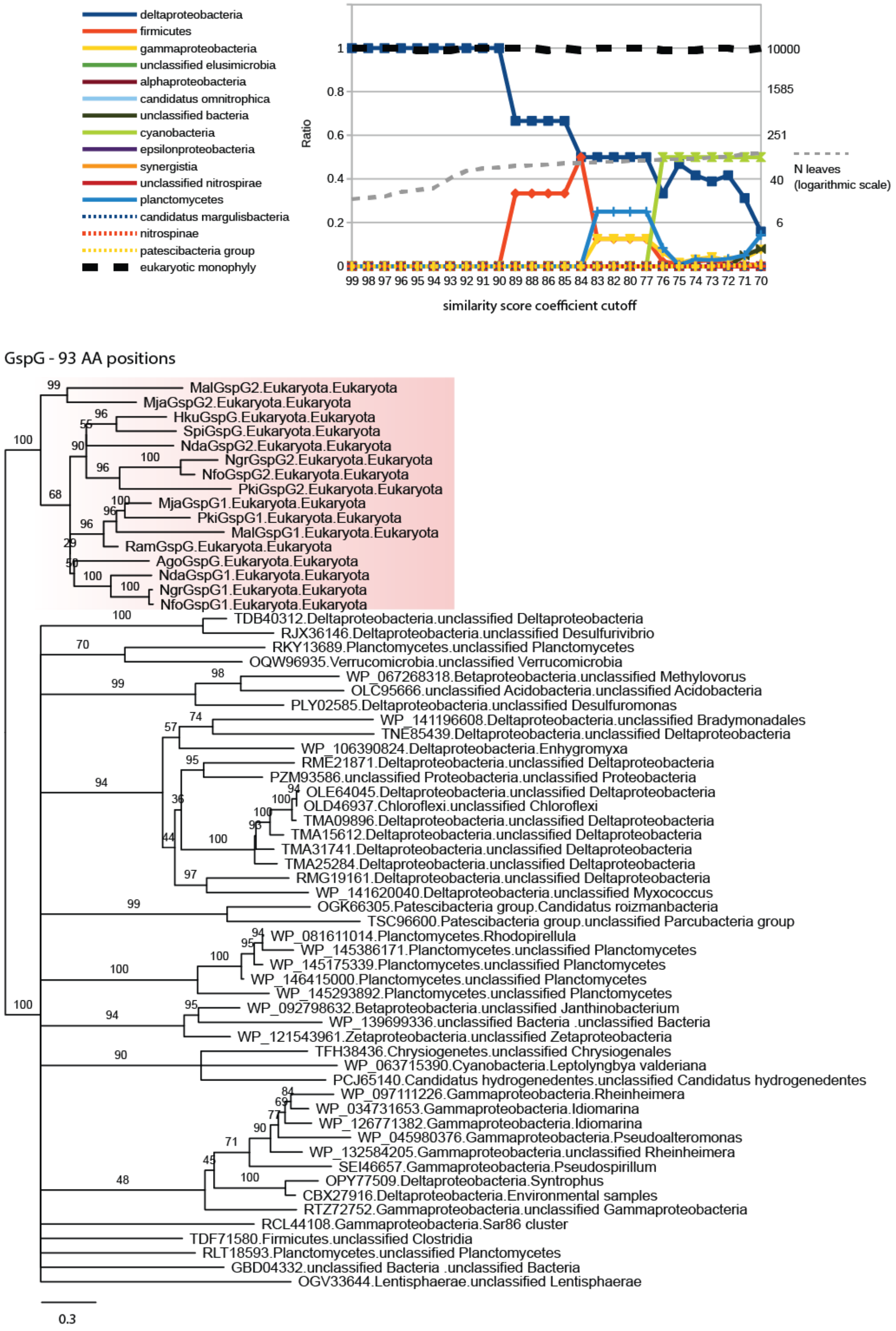

Supplementary Fig. 3D Phylogenetic relationship of the eukaryotic GspG proteins to prokaryotic homologues. For further explanations see page 7.

Supplementary Fig. 3 (pg. 3-6) Phylogenetic relationship of the eukaryotic GspD (panel A), GspE (panel B, pg. 4), GspF (panel C, pg. 5), and GspG (panel D, pg. 6) proteins to prokaryotic homologues. **Top part of each panel:** The monophyly of eukaryotic Gsp sequences and their relation to the prokaryotic homologues was systematically analyzed by building phylogenetic trees (using IQ-TREE) from gradually expanding datasets. Specifically, the different sets of sequences were defined by varying the HMMER score cutoff (in searches using profile HMMs built for the eukaryotic Gsp families) based on the formula $score_{cutoff} = c*score_{best\ prokaryotic\ hit}$, with the coefficient c decreasing from 0.99 to 0.70 incrementally by 0.01 (X axis). The resulting trees were systematically analyzed for support of the monophyly of eukaryotic sequences and for the taxonomic assignment of the parental prokaryotic node of the eukaryotic subtree (details of the procedure are described in Materials and Methods). The Y axis of the plot simultaneously denotes three variables: (i) support of the monophyly of eukaryotic sequences (black dashed line; percentages are rescaled to values 0 to 1); (ii) the taxonomic affiliation of the parental prokaryotic node of the eukaryotic subtree plotted as a fraction (0-1) for each prokaryotic taxonomic group (lines and shapes of a different colour, see the graphical legend to the right); and (iii) number of leaves (individual sequences) in the respective tree (grey dashed line; the values rescaled to their thousandth). **Bottom part of each panel:** Exemplar phylogenetic trees based on datasets defined by particular HMMER score cutoffs (dotted arrow). The trees were arbitrarily rooted between the clade of eukaryotic Gsp homologues (highlighted by a light red background) and prokaryotic sequences. The leaves are described accordingly: "organism name"."main taxon"."sequence ID"."system classification". The "system classification" has been assigned using the models of subunits of T4P superfamily of molecular machines[1]. Ultra-fast bootstrap values are shown at internal branches.

(A) GspD and GspDL

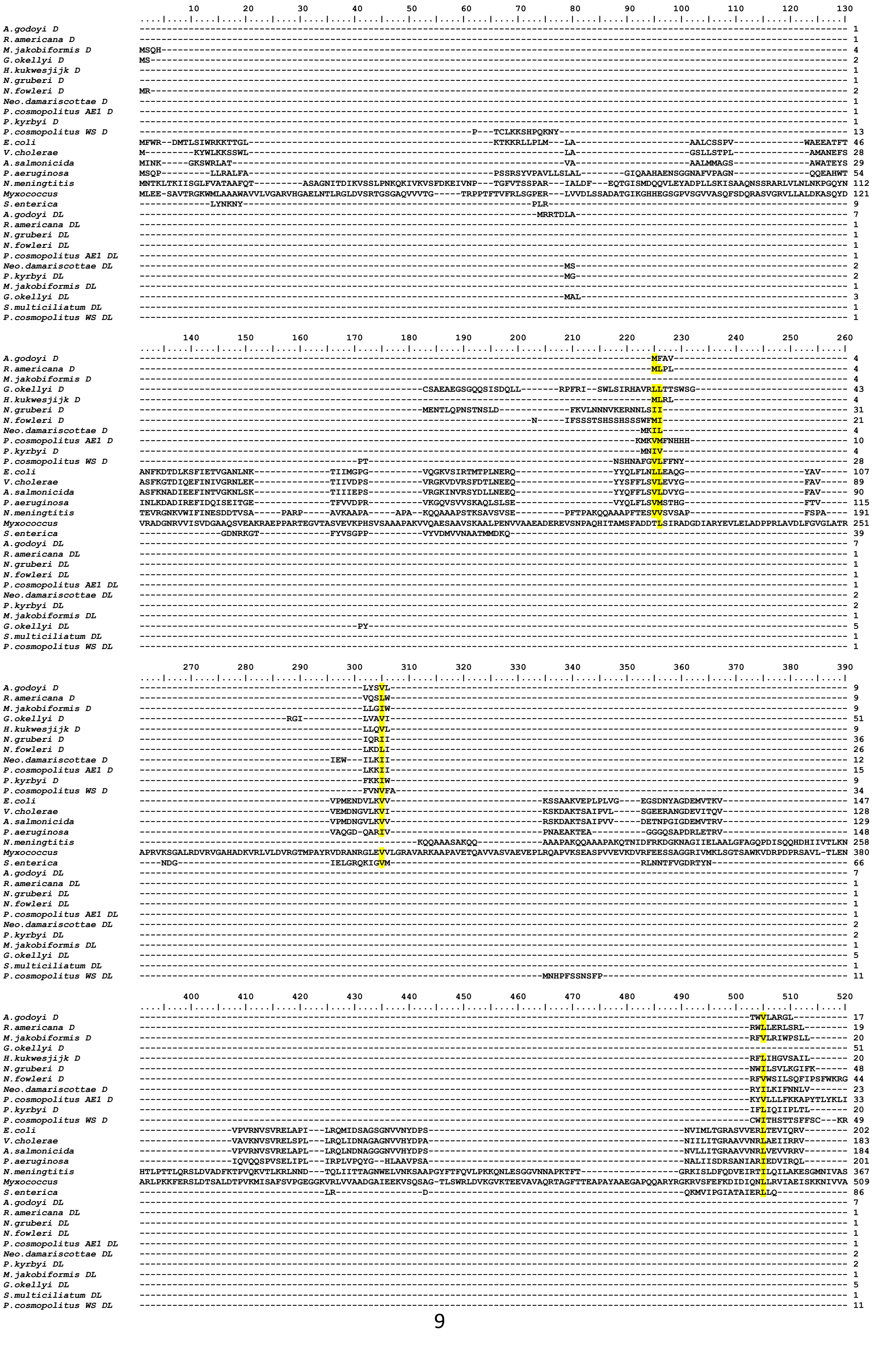

```
                        ....|....|....|....|....|....|....|....|....|....|....|....|....|....|....|....|....|....|....|....|....|....|....|....|....|....|
A.godoyi D              RLRRGT-----------------------------------------VCINERSFL----------CPVAAHGVAG--------------QAVESAAGLQTV-----TCTALE----------------- 60
R.americana D           -------------------------------------------WRWLLWKSGRR------------PERAAE-----------------SASTQSVRTVSAL-----EMQALEWSTS------------- 59
M.jakobiformis D        -------SGSIAAL-----------------------------ASWYIWRINRR------------APVVQPSE---------------EMPLTKAGQISAL-----NMAANAFADP------------- 69
G.okellyi D             -------------------------------------------AVVYFLLSRRRGH----------SPAALH------------------SVQAVEGGVSLL-----NAMANEFTGN------------- 92
H.kukwesjijk D          -------------------------------------------AQWRSIVSRLL------------FPVASHLSSSP-------------SHLVCSSSVTAL-----NLQANAFFSQ------------- 64
N.gruberi D             SNTSNNTNNNMFK-----------------------------------------------------SPISNSGDDDS------------NNTASSSFECSQL-----SIYSDLFNQQ------------- 95
N.fowleri D             RRVSNTTTITT-------------------------------------------------------TTTDFHNTSNTNST------TSSSLTTKQQQQHSQL-----EINSYLFSND------------- 95
Neo.damariscottae D     ------------------------------------------------------------------FPQQKQTETQE------------NETIIQRFEFSEL-----EYLSNQYSNR------------- 57
P.cosmopolitus AE1 D    NKVLDTCILKIFNIKTIQF-----------------------------------------------IPHSLHPFVDQTMKHPKKNIMIPNIIVPSKRNITNL-----EMFSQAFRAPEQ----------- 100
P.kyrbyi D              -------IYALFSL-----I----------------------GSILPVLSSTRKNH----------FPTKPH------------------NSSIQSKKVTGL-----EMAANQWNTG------------- 70
P.cosmopolitus WS D     RGVAGAGNYSVWFI--------------------------FCFSFFTWLRERRKSHGVAVKRNGVSLPR--------------------DSTRSNERDISEL-----ELLSFQYQDE------------- 115
E.coli                  -DHAGNRTEEVIPLDNASASEIARVLESLTKN---SGENQPATLKSQIVADERTNSVI------VSGDPATRDKMRRLIR-------RLDSEMERSGNS--------QVFYLKYSKAEDLVDVLKQVSGT 307
V.cholerae              -DQAGDKEIEVVELNNASAAEMVRIVEALNKT--TDAQNTPEFLKPKFVADERTNSIL------ISGDPKVRERLKRLIK-------QLDVEMAAKGNN--------RVVYLKYAKAEDLVEVLKGVSEN 289
A.salmonicida           -DKAGDQEVDIIKLKYASAGEMVRLVTNLNKDGNTQGGNTSLLLAPKVVADERTNSVV------VSGEPKARARIIQMVR-------QLDRDLQSQGNT--------RVFYLKYGKAKDMVEVLKGVSTS 292
P.aeruginosa            -DQKGSHDYSVINLRYGWVMDAAEVLNNAMSRGQAKGA-----AGAQVIADARTNRLI------ILGPPQARAKLVQLAQ-------SLDTPTARSANT--------RVIRLRHNDAKTLAETLGQISEG 304
N.meningtitis           DSVNGKMTLSLKDVPWDQALDLV--------------------MQARNLDMRQQGNIV------NIAPRDELLAKDK-------------AFLQAEKDIADLGALYSQNFQLKYKNVEEFRSILR----- 453
Myxococcus              DDVSGKVTIRLRNVPWDQALDLV--------------------LRTKALGKEEFGNII------RIAPLKTLEEEARLRQ-------ERKASLQRQEDLMV------NLIPVNYAVAADMAARVK----- 595
S.enterica              ------------------------------------GEEQPL------------GNIV------SSEPPAMPA---------------------FSANGE-------KGKAANYAGGMSLQEALK----- 129
A.godoyi DL             ---------------------------------------------------------------------------------------------------------------------------------- 7
R.americana DL          ----------------------------------------------------------------------------------------------MAWDRRN------TRNPSLY---------------- 14
N.gruberi DL            -----------------------------------------------MFNQQRNNY-------------------------------------MMGGGSGS-----GQIQPS-Y--M------------- 25
N.fowleri DL            ---------------------------------------------MSFQQQQRTNR-------------------------------------MMLPSSYQ------DMSPS-Y--L------------- 26
P.cosmopolitus AE1 DL   ----------------------------------------------MYQQQQPPGY-------------------------------------SYSMNPYN------QTAPS-Y---------------- 24
Neo.damariscottae DL    -------------------------------------------FYSQYGQNEPTNYGS-------------------GYG-------NNYGGSSYSGGGYG------GNAVG-YNRN------------- 43
P.kyrbyi DL            -------------------------------------------RLSRYGYDDI------------------------------------------------------ATLPS-Y---------------- 18
M.jakobiformis DL       -----------------------------------------------------------------------------------------------MDNVY------------------------------ 5
G.okellyi DL            -DRAG--------------------------------------AYGGYGGFGGPGYG---------GPGYGGAYGATGSG-------MGYGAGTYGTNGYGA----GGYGPSAYNTG------------- 63
S.multiciliatum DL      -------------------------------------------------------------------------------------------------------------MSS-Y---------------- 4
P.cosmopolitus WS DL    -----------------------------------------------------------------------------------------------------------PHQQSQYFPA------------- 21

                                660       670       680       690       700       710       720       730       740       750       760       770       780
                        ....|....|....|....|....|....|....|....|....|....|....|....|....|....|....|....|....|....|....|....|....|....|....|....|....|....|
A.godoyi D              ---------------------------------------------------------------------------------------------------------------------------------- 60
R.americana D           ---------------------------------------------------------------------------------------------------------------------------------- 59
M.jakobiformis D        ---------------------------------------------------------------------------------------------------------------------------------- 69
G.okellyi D             ---------------------------------------------------------------------------------------------------------------------------------- 92
H.kukwesjijk D          ---------------------------------------------------------------------------------------------------------------------------------- 64
N.gruberi D             ---------------------------------------------------------------------------------------------------------------------------------- 95
N.fowleri D             ---------------------------------------------------------------------------------------------------------------------------------- 95
Neo.damariscottae D     ---------------------------------------------------------------------------------------------------------------------------------- 57
P.cosmopolitus AE1 D    ---------------------------------------------------------------------------------------------------------------------------------- 100
P.kyrbyi D              ---------------------------------------------------------------------------------------------------------------------------------- 70
P.cosmopolitus WS D     ---------------------------------------------------------------------------------------------------------------------------------- 115
E.coli                  LTAAKEEAEGTVGSGRE-----VVSIAASKHSNALIVTAPQDIMQSLQSVIEQLDIRRAQVHVEALIVEVAEGSNINFGVQWGSK-----DAG----LMQFANGTQIPIGTLGAAISAAKPQKGSTVISE 423
V.cholerae              LQAEKGTGQPTTSKRN------EVMIAAHADTNSLVLTAPQDIMNAMLEVIGQLDIRRAQVLIEALIVEMAEGDGINLGVQWGSL-----ESG---SVIQYGN-TGASIGNVMIGLEEAKDTTQTKAVYD 404
A.salmonicida           IEADKKGGGATAGGGNASIGGGKLAISADETTNALVITAQPDVMAELEQVVAKLDIRRAQVLVEAIIVEIADGDGLNLGVQWANT-----NGG----GTQFTN-AGPGIGSVAIAAKDYKDN-------- 404
P.aeruginosa            MKNNGGQGGEQTGGGRPS----NILIRADESTNALVLLADPDTVNALEDIVRQLDVPRAQVLVEAAIVEISGDIQDAVGVQWAIN-----KGGMGGTKTNFAN-TGLSIGTLLQSLESNKAP-------- 416
N.meningtitis           ------LDNADTTGNRNTLVSGRGSVLIDPATNTLIVTDTRSVIEKFRKLIDELDVPAQQVMIEARIVEAADGFSRDLGVKFG-------ATGKK----KLKNDTSAFGWGVNSGFGGDDKW-------- 558
Myxococcus              -----------------DVLSERGSVTVDQRTNVLIVKDVRSNTERARSLVRSLDTQTPQVLIESRIVEANTSFSRSLGVQWGGQARAGQATGNS-TGLIFPNNLAVTGGVTGTGAGLPDN--------- 698
S.enterica              ----------------QNAAAGNIKIVAYPDTNSLLVKGTAEQVHFIEMLVKALDVAKRHVELSLWIVDLNKSDLERLGTSWSGSITIGDKLG------------------------------------- 206
A.godoyi DL             ---------------------------------------------------------------------------------------------------------------------------------- 7
R.americana DL          -----------------------------------------------------------------------------------------------------------DTR-------------------- 17
N.gruberi DL            ----------------------------------------------------------------------------------------------------YNDMISPSSS-------------------- 35
N.fowleri DL            ----------------------------------------------------------------------------------------------------YNDAPYGGYG-------------------- 36
P.cosmopolitus AE1 DL   ---------------------------------------------------------------------------------------------------------------------------------- 24
Neo.damariscottae DL    ----------------------------------------------------------------------------------------------------YMN--------------------------- 46
P.kyrbyi DL             ---------------------------------------------------------------------------------------------------------------------------------- 18
M.jakobiformis DL       ---------------------------------------------------------------------------------------------------------------------------------- 5
G.okellyi DL            ----------------------------------------------------------------------------------------------------YGSGGYGTTGGYGAGGYGPSGY-------- 85
S.multiciliatum DL      ---------------------------------------------------------------------------------------------------------------------------------- 4
P.cosmopolitus WS DL    ---------------------------------------------------------------------------------------------------------------------------------- 21

                                790       800       810       820       830       840       850       860       870       880       890       900       910
                        ....|....|....|....|....|....|....|....|....|....|....|....|....|....|....|....|....|....|....|....|....|....|....|....|....|....|
A.godoyi D              ---------------------------------------------------------------------------------------------------------------------------------- 60
R.americana D           -------------------------------------------------AVATVVDSDII-TLEEPE---------------------------------------------------EEPESSTAVALA 88
M.jakobiformis D        ---------------------------------------------------------------------------------------------------------------------------------- 69
G.okellyi D             --------------------------------------------------------------------------------------------------------------------AGTANLPALRLNLG 106
H.kukwesjijk D          ----------------------------------------------------QTAHHGHQQQLQPQT--AGLFSQLFHDSC---------------TSAQDLTTSREL---------------------- 103
N.gruberi D             ---------------------------------------------------VNIDDNHQIQSIKPNH----------------------------------INHDDKP---------QLMNQIDI----- 126
N.fowleri D             -------------------------------------------------HHPDIISSPSLFNTQPNV--VPLFSNHHQNHTTSVVGDDPHQLLTTFLIPMSNNVEFHK---------CDASSS----YLL 161
Neo.damariscottae D     ------------------------------------------------------------------------------------------------------------------------NENKSQLKMI 67
P.cosmopolitus AE1 D    ----------------------------------------------DHSQTADRFYQEVYETLLNPS------------------HKIKSSTDTTFKFPVDKNISLKD---------SLATQHPTIGEMS 157
P.kyrbyi D              ----------------------------------------------------SKTKQTINEDLENPK--LSSTNN---------------------THNNDLNLSSHP---------NEEVNV----QII 112
P.cosmopolitus WS D     ----------------------------------------------------NCVGEPHILTDNGNN--ATVVRNANFITAFVGVRDKVHTQGNSKVLMNHTNASNDPTSFNG----------------- 174
E.coli                  NGATTINPDTN---GDLSTLAQLLSGFSGTAVGVVKGDWMALVQAVKNDSSSNVLSTPSITTLDNQE--AFFMVG----------QDVPVLTGS--TVGSNNSNPFNT---------VERKKVGIMLKVT 527
V.cholerae              TNNNFLRNETTTTKGDYTKLASALSSIQGAAVSIAMGDWTALINAVSNDSSSNILSSPSITVMDNGE--ASFIVG----------EEVPVITGS--TAGSNNDNPFQT---------VDRKEVGIKLKVV 511
A.salmonicida           --------------GTTTGIAKLAESFNGMAAGFYQGNWAMLVTALSTNTKSDILSTPSIVTMDNKE--ASFNVG----------QEVPVQTGK--QNSTSGDTTFST---------IERKTVGTKLVVT 497
P.aeruginosa            -----------------------ESIPDGAIVGIGSSSFGALVTALSANTKSNLLSTPSLLTLDNQK--AEILVG----------QNVPFQTGSYTTNSEGSSNPFTT---------VERKDIGVSLKVT 502
N.meningtitis           GAETKINLPIT-----------AAANSISLVRAISSGALNLELSASESLSKTKTLANPRVLTQNRKE--AKIESG----------YEIPFTVTSIANGGSSTNTELKK--------------AVLGLTVT 651
Myxococcus              -PNFAVNLPTG------TGQGVGGAMGFTFGSAGGALQLNLRLSAAENEGSVKTISAPKVTTLDNNT--ARISQG----------VSIPFSQ----TSAQGVNTTFVE--------------ARLSLEVT 791
S.enterica              -----------------------VSLNQSSISTLDGSRFIAAVNALEEKKQATVVSRPVLLTQENVP--AIF----------------------------DNNRTFYTKLIGERNVALEHVTYGTMIRVL 283
A.godoyi DL             ---------------------------------------------VAERRSVESLLRDPLYNRSGRRWVSPYEDGRP-SLRRLDYAD----------------LPFE---------------------DR 54
R.americana DL          ---------------------------------------------ALMDPQAPYVAQ---------Q--RDRYTNMF-DPRSRYFED----------------FPYR---------------------ER 53
N.gruberi DL            ---------------------------------------------SMMRNNSPYMNSDPYYNPMSNR--RGYYDNYMIGNEPLYHED----------------KLFDEDLLMY---------------DR 87
N.fowleri DL            ---------------------------------------------GLSTPSPPYNYSDPYYNSMQNR--RGYYDNYMIGNEPLYHED----------------KLFDEDLLMY---------------DR 88
P.cosmopolitus AE1 DL   ----------------------------------------------FNRPQTRYAYQA--QPEYGGV---PYGSQYY-QQQPLYMED----------------RLFDDETAHY---------------AR 71
Neo.damariscottae DL    ---------------------------------------------RRNMPTSSYRRSP--YNQEGNM--SSYYDEYN-NNSPQYYED----------------RIYDDEAREY---------------ER 95
P.kyrbyi DL             ----------------------------------------------FRQP-TSYDY-----------------NRGR-NNTGRYTR------------------------------------------TR 41
M.jakobiformis DL       -------------------------------------------------------------PPPYFG--GDYYN----GFQPQWY--------------------------------------------- 23
G.okellyi DL            VPRYGGAGPMP----------------------------------GFVGP-QMYGPGPGQMPPLAGA--PGFFP----GFAPGW---------------------------------------------- 128
S.multiciliatum DL      ------------------------------------------------------------RDPPSHH--PGY------SNHPRYSSAGP-----------AAATPVSDSA------------------EW 37
P.cosmopolitus WS DL    ------------------------------------------------NSSNPYSWQSQYQNLPSHASPYSFYGGNAAANNYASPQD----------------QIFSNDEMQYI--------------NR 73

                                920       930       940       950       960       970       980       990      1000      1010      1020      1030      1040
                        ....|....|....|....|....|....|....|....|....|....|....|....|....|....|....|....|....|....|....|....|....|....|....|....|....|....|
A.godoyi D              ----------------------AASVAGVRRSRWQGMRIKKHFVTQTAVRVRP-SDSQFLGSFALSTELRTVQSGP-------LALIP----FL--KHWF--SRTEFVLVKSAILVFLTLHVEPYRPVLA 152
R.americana D           P------------------------QPTMGASRYSAIRIKKNFTLQTAMLLKE-NVPSYAGSFALATELRRVRRSP-------LSFIP----FL--ERFF--SRHEFMLVKSAILVFITVRTEAAGRPQV 178
M.jakobiformis D        ----------------------AVSMLASRRSRLVSMRIRKNFALQTSVMLKE-NQATYVGAFAISNELKTIRRGP-------LSYIP----LI--GDLF--RRDEFMRVKSAILVFVTCRAERTGSGPR 161
G.okellyi D             P--------------------------LALRSRLTSMRLRKNFTLQTSLMLKE-GQTTYAGSFALASELKTLKQGP-------LSFLP----VV--GQLF--SRDEFMLIKSAVLVFVTFHKERSQAAAA 194
H.kukwesjijk D          P---LVNAADCA---------FGDTFGGGGASRIESLRARKNFSLQTTMMLKE-NQTTYVGALAVGTELRTVKRSP-------LSYIP----FL--SRLF--TREEFMRVKSAILVFITARLQKLQPHVL 205
N.gruberi D             ----QFNKCI-----------SGTALPSMNSSKYEAMRIKKHFTTSTNLLIKEFDKPHFVGSFSNSTELKTIRKSP-------LSFIP----FL--SDLF--TRNEYMIVKSAILVFITASLKND----- 221
N.fowleri D             P----------------------SSLSPSSSSKYQAMRIKKHFTASTNLLIRELNTPHFVGSFANSTELKAIRKSP-------LSFLPF--QFL--SQLF--SRNEYMLVKSAILVFITASLDQQQQHDH 256
Neo.damariscottae D     P------------------------VSDELKSRWESMRLKKHFTFQTNVLLKP-NQPTYVGAFSSSTELQTVKRSP-------FSYLP----FL--RDLF--SRKEFILIKSAILVFITAKYEDKKTNQL 157
P.cosmopolitus AE1 D    PYGGLFN--------------PFTGFSELVKSSWNAMRLKKHFTVQTNMLLTP-NEPTFVGAFAVSQQLKTIKRSP-------LSFLP----FL--KWIF--ARTEYMLIKSAVLVFITARFSES----- 252
P.kyrbyi D              P-----------------------SNSNNGRSRWDAMRLKKHFAVQTTVMLKE-NEPSFAGAFAASTELRTVKKSP-------FSFLP----FKWGKALF--SRTEFMLVKSAILVFIKCIPQRAFNVTE 205
P.cosmopolitus WS D     PVGDFFSDPA-----------NPFSPVAYGKSSIQAIRLRKHFTCQTQLMVQP-NEPTYVGAFAMSTDIRTVRQSP-------LRALW--------GWLW--DRTEYLKIKSALLVFVTCRILKPDET-- 273
E.coli                  P---QINEGNAVQMVIEQEV-SKV----EGQTSLD--VVFGERKLKTTVLAND-GELIVLGGLMDDQAGESVAKVP------LLGDIP----LI--GNLF--KSTADKKEKRNLMVFIRPTILRDGMAAD 632
V.cholerae              P---QINEGNSVQLNIEQEV-SNV---LGANGAVD--VRFAKRQLNTSVMVQD-GQMLVLGGLIDERALESESKVP------LLGDIP----LL--GQLF--RSTSSQVEKKNLMVFIKPTIIRDGVTAD 617
A.salmonicida           P---QINEGDSVLLTIEQEV-SSVGKQASGTEGLG--PTFDTRTVKNAVLVKS-GETVVLGGLMDEQTKEEVSKVP------LLGDIP----VL--GYLF--RSTSNTTSKRNLMVFIRPTILRDANVYS 606
P.aeruginosa            P---HINDGAALRLEIEQEI-SALLPNAQQRNNTD--LITSKRSIKSTILAEN-GQVIVIGGLIQDDVSQAESKVP------LLGDIP----LL--GRLF--RSTKDTHTKRNLMVFLRPTVVRDSAGLA 611
N.meningtitis           P---NITPDGQIIMTVK----ITKDSPAQCASGNQTILCISTKNLNTQAMVEN-GGTLIVGGIYEEDNGNTLTKVP------LLGDIP----VI--GNLF--KTRGKKTDRRELLIFITPRIMGTAG--- 756
Myxococcus              P---HITQDGSVLMSIN----ASNNQPDPSSTGANGQPSIQRKEANTQVLVKD-GDTTVIGGIYVRRGATQVNSVP------FLSRIP----VL--GLLF--KNNSETDTRQELLIFITPRILNRQTIAQ 899
S.enterica              P---RFSADGQIEMSLDIEDGNDKTPQSDTTTSVDALPEVGRTLISTIARVPH-GKSLLVGGYTRDANTDTVQSIP------FLGKLP----LI--GSLF--RYSSKNKSNVVRVFMIEPKEIVDPLTPD 395
A.godoyi DL             P--------------------TYVPSQLLANPDFGNFRIRRSFT--TELWLQD-GETVAVLGDGKEREFRT-HYSPMYRRPFFSGWIGRLLQFLALDRLFGLHIDSHKKSRTRSVLLVTAKLLPLNEQRT 160
R.americana DL          A--------------------TYSRGSALGNPDFGNFRFRKGYS--AELTLRD-GETAMLSGDSRSKSYRT-HYSPEYRSSFFYSWLGKLMRLLWLDRLFGLHVDTVKHVRTRSYVFVTARILPDAATAT 159
N.gruberi DL            P--------------------TYRPSRMIANPDFGNFNYKKGYT--AEVWLND-GETVVLSGDSRKKTYRT-HYSPEYLRSFFFGWVGRLIRYLWMDRLFGLHVDTVKEVKTKNYIFVTAQLLSE----- 188
N.fowleri DL            P--------------------TYRPSRMIANPDFGNFNYKKGFT--AEVWLND-GETVVLNGDSRKKTYRT-HYSPEYLRSFFFGWVGRLIRYLWLDRLFGLHVDTVKEVKTRNYIFVTTQIIPE----- 189
P.cosmopolitus AE1 DL   P--------------------SYRRSAMLGNPDFGNYHFKKGYA--TEVWLND-GETVVLAADTRKKKYRT-HYSPQFLRSFFFGWIGRLLRFLMLDRLFGLHVDTTKTVTTRNYIFVTAHVLPS----- 172
Neo.damariscottae DL    P--------------------TYRRSKTIANPDFGNFHFKKGYT--AEVWLND-GETVVLSSDSRKKSYRT-HYSPEYMRSFFFGWIGRLLRFFYLDRLFGLHVDTVKNVTIKNFIFVTAHSLPI----- 196
P.kyrbyi DL             P--------------------TYVRSNLAASPDFGNFGFRKGYT--ASVWMND-GETVVLSADRKQRTYRT-HYSPEFLRSFFYGWLGRLLRFFMLDRLFGFHMDTVKTVNKRSFVFVTAHVLDT----- 142
M.jakobiformis DL       ----------------------YRPSRLLANPDFGNFRYKKGFT--AEVTLED-GETVVLAADSRVKTYRT-HYSP--FNRYSFSWLDRILRFLWLDRLLGLNIDSVKRIRTKSFIFVTAHLLPH----- 120
G.okellyi DL            ---------------------AYRPASLAANPDFGTYRYKKGYT--AEVTLQD-GETVVLAADSRTKSYRT-QYSP--FNRYSFSWLDRLLRFLWLDRLLGLHVDSVKHVRTKSFICVTAHLLPS----- 226
S.multiciliatum DL      P--------------------QPRTSVLQGSPDFGNLHFRKGFT--SEVMLND-GETAILSAHSRNRSYRL-RYSPIF--SFVYGWIRRFLAFFWLDRLLGPQVDSVKTVKTKSFVMVSAHILPAV---- 137
P.cosmopolitus WS DL    P--------------------TMHRSSLLASPSFPNLSLKHAIS--TNVTLES-GRTVALQSQEREKVFRG-RYWSGWKWYRMSGWLKVLVYAF--RWLIGLNIDTVKKVKVSNMIYLTGYVLPT----- 172
```

```
                                1050      1060      1070      1080      1090      1100      1110
                         ....|....|....|....|....|....|....|....|....|....|....|....|....|....|....|...
A.godoyi D               LNDIAEQ-----IED--------------------------------------------------------------- 162
R.americana D            TQS--------------------------------------------------------------------------- 181
M.jakobiformis D         LTQQQQQ---PMLQTDARY----------------------------------------------------------- 177
G.okellyi D              PVQTAL------------------------------------------------------------------------ 200
H.kukwesjijk D           AQ---------------------------------------------------------------------------- 207
N.gruberi D              ------------------------------------------------------------------------------ 221
N.fowleri D              NNMNNTSIGSSSISGGHGSGG--------------------------------------------------------- 277
Neo.damariscottae D      KGNVIKK----------------------------------------------------------------------- 164
P.cosmopolitus AE1 D     ------------------------------------------------------------------------------ 252
P.kyrbyi D               TTM-------PAIENEPQYFGEQ-----------------Y------------------------------------- 222
P.cosmopolitus WS D      ------------------------------------------------------------------------------ 273
E.coli                   GVSQRKY---NYMRAEQIYRDEQ-GL-SLMPHTAQPILP--AQNQA-LPPEVRAFLNAGRTR---------------- 686
V.cholerae               GITQRKY---NYIRAEQLFRAEK-GL-RLLDDASVPVLPKFGDDRR-HSPEIQAFIEQMEAKQ--------------- 674
A.salmonicida            GISSNKY---TLFRAQQLEAAAQKGY-ATSPD--RQVLPEYGQDVV-QSPEIQKQIELMKARQQATADGAQPFVQGNN 677
P.aeruginosa             ALSGKKY---SDIRVIDGTRGPE-GRPSILPTNANQLFDGQAVDLRELMTE--------------------------- 658
N.meningtitis            ----------NSLRY--------------------------------------------------------------- 761
Myxococcus               TL---------------------------------------------------------------------------- 901
S.enterica               ASESVN----NILKQSGAWSGDD-----------------------KLQKWVRVYLDRGQEAI--------------K 432
A.godoyi DL              ATDKSN----DDVSVSES------------------------------------------------------------ 174
R.americana DL           AAPSQA----SEVS---------------------------------------------------------------- 169
N.gruberi DL             ------------------------------------------------------------------------------ 188
N.fowleri DL             ------------------------------------------------------------------------------ 189
P.cosmopolitus AE1 DL    ------------------------------------------------------------------------------ 172
Neo.damariscottae DL     ------------------------------------------------------------------------------ 196
P.kyrbyi DL              ------------------------------------------------------------------------------ 142
M.jakobiformis DL        ------------------------------------------------------------------------------ 120
G.okellyi DL             ------------------------------------------------------------------------------ 226
S.multiciliatum DL       ------------------------------------------------------------------------------ 137
P.cosmopolitus WS DL     ------------------------------------------------------------------------------ 172
```

(B) GspDN1

```
AgoGspDN1      ---------------------------------------------------------------------------------------------------------------------------------- 1
RamGspDN1      ---------------------------------------------------------------------------------------------------------------------------------- 1
NgrGspDN1      ---------------------------------------------------------------------------------------------------------------------------------- 1
NfoGspDN1      ---------------------------------------------------------------------------------------------------------------------------------- 1
NdaGspDN1      ---------------------------------------------------------------------------------------------------------------------------------- 1
PcoAEGspDN1    ---------------------------------------------------------------------------------------------------------------------------------- 1
PkiGspDN1      ---------------------------------------------------------------------------------------------------------------------------------- 1
GokGspDN1      ---------------------------------------------------------------------------------------------------------------------------------- 1
SpiGspDN1      ---------------------------------------------------------------------------------------------------------------------------------- 1
E.coli         MFWR--------DMT----------------------------------------------LSIWRKKTTGLKTKKR-LLPLMLAA-----------------------ALCSSPVWAEEATFTANFKDT 52
V.cholerae     -------------------------------------------------------------MKYWLKKSSWL-----------LAG-----------------------SLLSTPL-AMANEFSASFKGT 34
A.salmonicida  ---------------------------------------------------------------MINKGKSWR-------LAT-VAA-----------------------ALMMAGS-AWATEYSASFKNA 35
P.aeruginosa   MSQP--------LLR----------------------------------------------ALFAPSSRSYVPAVLL-SLALGIQAAH--------------AENSGGNAFVPAGN-QQEAHWTINLKDA 60
N.meningtitis  MNTK--------LTKIISGLFVATAAFQTASAGNITDIKVSSLPNKQKIVKVSFDKEIVNPTGFVTSSPARIALDF---EQTGISMDQQVLEYADPLLSKISAAQNSSRARLVLNL-NKPGQYNTEVRGN 118
Myxococcus     MLEESAVTRGKWMLAAAWAVVLVGARVHGAELNTLRGLDVSRTGSGAQVVVTGTRPPTF--TVFRLSGPERLVVDLSSADATGIKGHHEGSGPVSGVVASQFSDQRASVGRVLLAL-DKASQYDVRADGN 127
S.enterica     LYNKNYPLRGD----------------------------------------------------------------------------------------------------------NRKGTF------- 17

                       140       150       160       170       180       190       200       210       220       230       240       250       260
               ....|....|....|....|....|....|....|....|....|....|....|....|....|....|....|....|....|....|....|....|....|....|....|....|....|....|
AgoGspDN1      ---------------------------------------------------------------------------------------------------------------------------------- 1
RamGspDN1      ---------------------------------------------------------------------------------------------------------------------------------- 1
NgrGspDN1      ---------------------------------------------------------------------------------------------------------------------------------- 1
NfoGspDN1      ---------------------------------------------------------------------------------------------------------------------------------- 1
NdaGspDN1      ---------------------------------------------------------------------------------------------------------------------------------- 1
PcoAEGspDN1    ---------------------------------------------------------------------------------------------------------------------------------- 1
PkiGspDN1      ---------------------------------------------------------------------------------------------------------------------------------- 1
GokGspDN1      ---------------------------------------------------------------------------------------------------------------------------------- 1
SpiGspDN1      ---------------------------------------------------------------------------------------------------------------------------------- 1
E.coli         DLKSFIETVGANLNK--------------TIIMGPG----------VQGKVSIRTMTPLNER------------------QYYQLFLNLLEAQG----------------------YAV----------- 107
V.cholerae     DIQEFINIVGRNLEK--------------TIIVDPS----------VRGKVDVRSFDTLNEE------------------QYYSFFLSVLEVYG----------------------FAV----------- 89
A.salmonicida  DIEEFINTVGKNLSK--------------TIIIEPS----------VRGKINVRSYDLLNEE------------------QYYQFFLSVLDVYG----------------------FAV----------- 90
P.aeruginosa   DIREFIDQISEITGE--------------TFVVDPR----------VKGQVSVVSKAQLSLS------------------EVYQLFLSVMSTHG----------------------FTV----------- 115
N.meningtitis  KVWIFINESDDTVSA-----PAR-----PAVKAAPAA-------PAKQQAAAPSTKSAVSVSE---------PFTPAKQQAAAPFTESVVSVSAP---------------------FSP----------- 190
Myxococcus     RVVISVDGAAQSVEAKRAEPPARTEGVTASVEVKPHSVSAAAPAKVVQAESAAVSKAALPENVVAAEADEREVSNPAQHITAMSFADDTLSIRADGDIARYEVLELADPPRLAVDLFGVGLATRAPRVKS 257
S.enterica     ---------------------------------------------------------------------------------------------------------------------------------- 17

                       270       280       290       300       310       320       330       340       350       360       370       380       390
               ....|....|....|....|....|....|....|....|....|....|....|....|....|....|....|....|....|....|....|....|....|....|....|....|....|....|
AgoGspDN1      ---------------------------------------------------------------------------------------------------------------------------------- 1
RamGspDN1      ---------------------------------------------------------------------------------------------------------------------------------- 1
NgrGspDN1      ---------------------------------------------------------------------------------------------------------------------------------- 1
NfoGspDN1      ---------------------------------------------------------------------------------------------------------------------------------- 1
NdaGspDN1      ---------------------------------------------------------------------------------------------------------------------------------- 1
PcoAEGspDN1    ---------------------------------------------------------------------------------------------------------------------------------- 1
PkiGspDN1      ---------------------------------------------------------------------------------------------------------------------------------- 1
GokGspDN1      ---------------------------------------------------------------------------------------------------------------------------------- 1
SpiGspDN1      ---------------------------------------------------------------------------------------------------------------------------------- 1
E.coli         -----------------------------VPMENDVLKVV----------------------------KSSAAKVEPLPLVG----EGSDNYAGDEMVTKV----------------------------- 147
V.cholerae     -----------------------------VEMDNGVLKVI----------------------------KSKDAKTSAIPVL-----SGEERANGDEVITQV----------------------------- 128
A.salmonicida  -----------------------------VPMDNGVLKVV----------------------------RSKDAKTSAIPVV-----DETNPGIGDEMVTRV----------------------------- 129
P.aeruginosa   -----------------------------VAQGDQA-RIV----------------------------PNAEAKTEA----------GGGQSAPDRLETRV----------------------------- 148
N.meningtitis  ----------------------------------------A----KQQAAASAKQQ------------AAAPAKQQAAAPAKQTNIDFRKDGKNAGIIELAALGFAGQPDISQQHDHIIVTLKNHTLPTT 264
Myxococcus     GALRDVRVGAHADKVRLVLDVRGTMPAYRVDRANRGLEVVLGRAVARKAAPAVETQAVVASVAEVEPLRQAPVKSEASPVVEVKDVRFEESSAGGRIVMKLSGTSAWKVDRPDPRSAVL-TLENARLPKK 386
S.enterica     ---------------------------------------------------------------------------------------------------------------------------------- 17

                       400       410       420       430       440       450       460       470       480       490       500       510       520
               ....|....|....|....|....|....|....|....|....|....|....|....|....|....|....|....|....|....|....|....|....|....|....|....|....|....|
AgoGspDN1      ---------------------------------------------------------------------------------------------------------------------------------- 1
RamGspDN1      ---------------------------------------------------------------------------------------------------------------------------------- 1
NgrGspDN1      ---------------------------------------------------------------------------------------------------------------------------------- 1
NfoGspDN1      ---------------------------------------------------------------------------------------------------------------------------------- 1
NdaGspDN1      ---------------------------------------------------------------------------------------------------------------------------------- 1
PcoAEGspDN1    ---------------------------------------------------------------------------------------------------------------------------------- 1
PkiGspDN1      ---------------------------------------------------------------------------------------------------------------------------------- 1
GokGspDN1      ---------------------------------------------------------------------------------------------------------------------------------- 1
SpiGspDN1      ---------------------------------------------------------------------------------------------------------------------------------- 1
E.coli         -----------VPVRNVSVRELAPI---LRQMIDSAGSGN-VVNYDP-----------------------------------------------SNVIMLTGRASVVERLTEVIQRV---------DHAG 206
V.cholerae     -----------VAVKNVSVRELSPL---LRQLIDNAGAGN-VVHYDP-----------------------------------------------ANIILITGRAAVVNRLAEIIRRV---------DQAG 187
A.salmonicida  -----------VPVRNVSVRELAPL---LRQLNDNAGGGN-VVHYDP-----------------------------------------------SNVLLITGRAAVVNRLVEVVRRV---------DKAG 188
P.aeruginosa   -----------IQVQQSPVSELIPL---IRPLVPQ--YGH-LAAVPS-----------------------------------------------ANALIISDRSANIARIEDVIRQL---------DQKG 205
N.meningtitis  LQRSLDVADFKTPVQKVTLKRLNND---TQLIITTAGNWE-LVNKSAAPGYFTFQVLPKKQNLESGGVNNAPKTF------------------TGRKISLDFQDVEIRTILQILAKESGMNIVASDSVNG 372
Myxococcus     FERSLDTSALDTPVKMISAFSVPGEGGKVRLVVAADGAIEEKVSQSA--GTLSWRLDVKGVKTEEVAVAQRTAGFTTEAPAYAAEGAPQQARYRGKRVSFEFKDIDIQNLLRVIAEISKKNIVVADDVSG 514
S.enterica     -----------------------------------------YVSGPP-----------------------------------------------VYVDMVVNAATMMDKQNDGIE-------------LG 46

                       530       540       550       560       570       580       590       600       610       620       630       640       650
               ....|....|....|....|....|....|....|....|....|....|....|....|....|....|....|....|....|....|....|....|....|....|....|....|....|....|
AgoGspDN1      -------------------------------------------------------------------------------------------MSNGPHYLFLQHSRLTDEVQKYVSSFVQYIASIVEPD-- 37
RamGspDN1      ---------------------------------------M-STPPAAAAAPPSVSPPPPLVTSTEAPPPDEDSAPASAPPLRRPVGDAAEGDTREPYYIFLQNTTLTRAVQRYIAAFVQHVAGVLEPEPP 90
NgrGspDN1      ----------------------------------------------MSKQQTTTNTLPT-----TST--------------------SEDDNIREPFYIYLQNSELNTNLQKYISAFVQHIASILEQD-- 57
NfoGspDN1      ----------------------------------------------MSTNQEDLTQRP------NKT--------------------DHDDDIREPFYIYLQNSELNTNLQKYISAFVQHIASILEQD-- 56
NdaGspDN1      --------------------------------------------------------------MNE----------------------GQQKQEKEPFYIYLENSQLNEKLQKYISDFIQHIASITEKE-- 44
PcoAEGspDN1    ----------------------------------------------------MTDSSPNPTQQEENE---------------------SKTQSREPYYIYLQNSELTEALQKYIAAFVQHIASILEKD-- 55
PkiGspDN1      -----------------------------------------MNSSSSSSESQFENNIPPPPNVNENALKD----------------IQLDNESREPFYIYLQNSILTNDLQRYIGAFVQHIASILEKN-- 71
GokGspDN1      -----------------------------------------------------------------------------------------MASAPEPYYIYLTNSVLDGNLQKYISAYVEHIAKTLEGP-- 39
SpiGspDN1      --------------------------------------------------------MLMDPLQDRSDQPQP----------------LGSEQPSAEPFCVHLSNAVMDPQLQQFISSFVDYIASVLEPP- 57
E.coli         NRTEEVIPLDNASASEIARVLESLTKNS---GENQPATLKSQIVADERTNSVIVSGDPATRDKMRRLIRRLDSEMERSGNS----------QVFYLKYSKAEDLVDVLKQVSGTLTAAKEEAEGTVGSGR 323
V.cholerae     DKEIEVVELNNASAAEMVRIVEALNKTTD--AQNTPEFLKPKFVADERTNSILISGDPKVRERLKRLIKQLDVEMAAKGNN----------RVVYLKYAKAEDLVEVLKGVSENLQAEKGTGQPTT-SKR 304
A.salmonicida  DQEVDIIKLKYASAGEMVRLVTNLNKDGNTQGGNTSLLLAPKVVADERTNSVVVSGEPKARARIIQMVRQLDRDLQSQGNT----------RVFYLKYGKAKDMVEVLKGVSTSIEADKKGGGATAGGGN 308
P.aeruginosa   SHDYSVINLRYGWVMDAAEVLNNAMSRGQAKGA-----AGAQVIADARTNRLIILGPPQARAKLVQLAQSLDTPTARSANT----------RVIRLRHNDAKTLAETLGQISEGMKNNGGQGGEQTGGGR 320
N.meningtitis  KMTLSLKDVPWDQALDLV--------------------MQARNLDMRQQGNIVNIAP------RDELLAKDKAFLQAEKDIA-D-LGALYSQNFQLKYKNVEEFRSILRLDNADTTGNRN----TLVSGR 470
Myxococcus     KVTIRLRNVPWDQALDLV--------------------LRTKALGKEEFGNIIRIAPLKTLEEEARLRQERKASLQRQEDLMV--------NLIPVNYAVAADMAARVK---------------DVLSER 601
S.enterica     RQKIGVMRLNNTFVGDRT--------------------------YNLRDQKMVIPGIATAIERLLQGEEQPLGNIVSSEPPAMPAFSANGEKGKAANYAGGMSLQEALKQ--------------NAAAGN 136

                       660       670       680       690       700       710       720       730       740       750       760       770       780
               ....|....|....|....|....|....|....|....|....|....|....|....|....|....|....|....|....|....|....|....|....|....|....|....|....|....|
AgoGspDN1      ---TEPSRRIISFLPDRNGVVLVGFQDSTVRVIHRFLEKMDEEKRTLKFTVHLVLAESHRMTSNFSF--------------------------------------------------------------- 101
RamGspDN1      GT--PSERRIVSFLPERNGVLFLGFRRSTVRVIYKFLTRMDTEKRNLHFEVNLVLVESHRMTSQFSF--------------------------------------------------------------- 155
NgrGspDN1      ---APNIRRIVSFLPERNGILLFGFRRSTIKVLYSFLKSLDKKKNNINFEINLVLVESHKMTNNFNF--------------------------------------------------------------- 121
NfoGspDN1      ---APNVRRIVSFLPERNGVLLFGFRRSTIKVLYSFLKSLDQKKHNINFEINLVLVESHKMTHDFNF--------------------------------------------------------------- 120
NdaGspDN1      ---ETTTKRVISFLPERNGILLLGFKSSTVKVIYHFLKKLDEEKRNINFEVNLVLVESHKMTSGFKF--------------------------------------------------------------- 108
PcoAEGspDN1    ---APSVKRIVSFLPERNGILLFGFQSTTIETIYRFLKGLDEKKYNIDFEVNLVLVESHKMTSGFKF--------------------------------------------------------------- 119
PkiGspDN1      ---DKNSRRVVSFLPERNGILLFGFKVSTVKVIYKFLRRLDEEKHNLHFEVNLVLVESHKMTGGFQF--------------------------------------------------------------- 135
GokGspDN1      ----AHANRVVTYIPERNGILLFGFQPETIRVIYRLLQKLDEETFNLRFKVDLVLVESHRMTSNFEF--------------------------------------------------------------- 102
SpiGspDN1      ---ESAEMRHVAFMPERGGILLFGFRPSTVQVIYRFLAKIDEERHNIRFEINLVYVESHKMTNAFEF--------------------------------------------------------------- 121
E.coli         -EVV-----SIAASKHSNALIVTA-PQDIMQSLQSVIEQLDIRRAQVHVEALIVEVAEGSNINFGVQWGSK-----DAG----LMQFANGTQIPIGTLGAAISAAKPQKGSTVISENGATTINPDTN--- 434
V.cholerae     -NEV-----MIAAHADTNSLVLTA-PQDIMNAMLEVIGQLDIRRAQVLIEALIVEMAEGDGINLGVQWGSL-----ESG---SVIQYGN-TGASIGNVMIGLEEAKDTTQTKAVYDTNNNFLRNETTTTK 418
A.salmonicida  -ASIGGGKLAISADETTNALVITA-QPDVMAELEQVVAKLDIRRAQVLVEAIIVEIADGDGLNLGVQWANT-----NGG----GTQFTN-AGPGIGSVAIAAKDYKDN---------------------- 404
P.aeruginosa   PSNI-----LIRADESTNALVLLA-DPDTVNALEDIVRQLDVPRAQVLVEAAIVEISGDIQDAVGVQWAIN-----KGGMGGTKTNFAN-TGLSIGTLLQSLESNKAP---------------------- 416
N.meningtitis  ------G--SVLIDPATNTLIVTD-TRSVIEKFRKLIDELDVPAQQVMIEARIVEAADGFSRDLGVKFG-------ATG----KKKLKN-DTSAFGWGVNSGFGGDDK--------WGAETKINLPI--- 568
Myxococcus     ------G--SVTVDQRTNVLIVKD-VRSNTERARSLVRSLDTQTPQVLIESRIVEANTSFSRSLGVQWGGQARAGQATG-NSTGLIFPN-NLAVTGGVTGTGAGLPDN----------PNFAVNLPT--- 707
S.enterica     ---I-----KIVAYPDTNSLLVKG-TAEQVHFIEMLVKALDVAKRHVELSLWIVDLNKSDLERLGTSWSGSITIGDKLG--------------------------------------------------- 206
```

```
                       790       800       810       820       830       840       850       860       870       880       890       900       910
               ....|....|....|....|....|....|....|....|....|....|....|....|....|....|....|....|....|....|....|....|....|....|....|....|....|....|
AgoGspDN1      ---------------------------------------------------------------------------------------------------------------------------------- 101
RamGspDN1      ---------------------------------------------------------------------------------------------------------------------------------- 155
NgrGspDN1      ---------------------------------------------------------------------------------------------------------------------------------- 121
NfoGspDN1      ---------------------------------------------------------------------------------------------------------------------------------- 120
NdaGspDN1      ---------------------------------------------------------------------------------------------------------------------------------- 108
PcoAEGspDN1    ---------------------------------------------------------------------------------------------------------------------------------- 119
PkiGspDN1      ---------------------------------------------------------------------------------------------------------------------------------- 135
GokGspDN1      ---------------------------------------------------------------------------------------------------------------------------------- 102
SpiGspDN1      ---------------------------------------------------------------------------------------------------------------------------------- 121
E.coli         GDLSTLAQLLSGFSGTAVGVVKGDWMALVQAVKNDSSSNVLSTPSITTLDNQEAFFMVGQDVPVLTGS--TVGSNNSNPFNTVERKKVGIMLKVTPQINEGNAVQMVIEQEV-SKV---EGQT-SLD--V 555
V.cholerae     GDYTKLASALSSIQGAAVSIAMGDWTALINAVSNDSSSNILSSPSITVMDNGEASFIVGEEVPVITGS--TAGSNNDNPFQTVDRKEVGIKLKVVPQINEGNSVQLNIEQEV-SNV---LGANGAVD--V 540
A.salmonicida  GTTTGIAKLAESFNGMAAGFYQGNWAMLVTALSTNTKSDILSTPSIVTMDNKEASFNVGQEVPVQTGK--QNSTSGDTTFSTIERKTVGTKLVVTPQINEGDSVLLTIEQEV-SSVGKQASGTEGLG--P 529
P.aeruginosa   ---------ESIPDGAIVGIGSSSFGALVTALSANTKSNLLSTPSLLTLDNQKAEILVGQNVPFQTGSYTTNSEGSSNPFTTVERKDIGVSLKVTPHINDGAALRLEIEQEI-SALLPNAQQRNNTD--L 534
N.meningtitis  -----TAAANSISLVRAIS--SGALNLELSASESLSKTKTLANPRVLTQNRKEAKIESGYEIPFTVTSIANGGSSTNTEL-----KKAVLGLTVTPNITPDGQIIMTVKITK-DSPAQCASGN-QTI--L 682
Myxococcus     GTGQGVGGAMGFTFGSAGG--ALQLNLRLSAAENEGSVKTISAPKVTTLDNNTARISQGVSIPFSQTS----AQGVNTTF-----VEARLSLEVTPHITQDGSVLMSINASN-NQPDPSSTGA-NGQ--P 822
S.enterica     ---------VSLNQSSISTLDGSRFIAAVNALEEKKQATVVSRPVLLTQENVPAIFDNNRTFYTKLIG------ERNVAL---EHVTYGTMIRVLPRFSADGQIEMSLDIEDGNDKTPQSDTTTSVDALP 318

                       920       930       940       950       960       970       980       990      1000      1010      1020      1030      1040
               ....|....|....|....|....|....|....|....|....|....|....|....|....|....|....|....|....|....|....|....|....|....|....|....|....|....|
AgoGspDN1      ---------------------------------------------------------------------------------------------------------------------------------- 101
RamGspDN1      ---------------------------------------------------------------------------------------------------------------------------------- 155
NgrGspDN1      ---------------------------------------------------------------------------------------------------------------------------------- 121
NfoGspDN1      ---------------------------------------------------------------------------------------------------------------------------------- 120
NdaGspDN1      ---------------------------------------------------------------------------------------------------------------------------------- 108
PcoAEGspDN1    ---------------------------------------------------------------------------------------------------------------------------------- 119
PkiGspDN1      ---------------------------------------------------------------------------------------------------------------------------------- 135
GokGspDN1      ---------------------------------------------------------------------------------------------------------------------------------- 102
SpiGspDN1      ---------------------------------------------------------------------------------------------------------------------------------- 121
E.coli         VFGERKLKTTVLANDGELIVLGGLMDDQAGESVAKVPLLGDIPLIGNLFKSTADKKEKRNLMVFIRPTILRDGMAADGVSQRKYNYMRAEQIYRDEQ-GLSLMPHTAQPILP--AQNQALPPEVRAFLNA 682
V.cholerae     RFAKRQLNTSVMVQDGQMLVLGGLIDERALESESKVPLLGDIPLLGQLFRSTSSQVEKKNLMVFIKPTIIRDGVTADGITQRKYNYIRAEQLFRAEK-GLRLLDDASVPVLPKFGDDRRHSPEIQAFIEQ 669
A.salmonicida  TFDTRTVKNAVLVKSGETVVLGGLMDEQTKEEVSKVPLLGDIPVLGYLFRSTSNTTSKRNLMVFIRPTILRDANVYSGISSNKYTLFRAQQLEAAAQKGYATSPDRQ--VLPEYGQDVVQSPEIQKQIEL 657
P.aeruginosa   ITSKRSIKSTILAENGQVIVIGGLIQDDVSQAESKVPLLGDIPLLGRLFRSTKDTHTKRNLMVFLRPTVVRDSAGLAALSGKKYSDIRVIDGTRGPE-GRPS-------ILPTNANQLFDGQAVDLR-EL 655
N.meningtitis  CISTKNLNTQAMVENGGTLIVGGIYEEDNGNTLTKVPLLGDIPVIGNLFKTRGKKTDRRELLIFITPRIMGTAG----------NSLRY---------------------------------------- 761
Myxococcus     SIQRKEANTQVLVKDGDTTVIGGIYVRRGATQVNSVPFLSRIPVLGLLFKNNSETDTRQELLIFITPRILNRQTIAQTL--------------------------------------------------- 901
S.enterica     EVGRTLISTIARVPHGKSLLVGGYTRDANTDTVQSIPFLGKLPLIGSLFRYSSKNKSNVVRVFMIEPKEIVDPLTPDA-SESVNNILKQSGAWSGDD--------------------KLQKWVRVYLDR 426

                      1050      1060
               ....|....|....|....|
AgoGspDN1      -------------------- 101
RamGspDN1      -------------------- 155
NgrGspDN1      -------------------- 121
NfoGspDN1      -------------------- 120
NdaGspDN1      -------------------- 108
PcoAEGspDN1    -------------------- 119
PkiGspDN1      -------------------- 135
GokGspDN1      -------------------- 102
SpiGspDN1      -------------------- 121
E.coli         GRTR---------------- 686
V.cholerae     MEAKQ--------------- 674
A.salmonicida  MKARQQATADGAQPFVQGNN 677
P.aeruginosa   MTE----------------- 658
N.meningtitis  -------------------- 761
Myxococcus     -------------------- 901
S.enterica     GQEAIK-------------- 432
```

(C) GspDN2

```
                       10        20        30        40        50        60        70        80        90       100       110       120       130
               ....|....|....|....|....|....|....|....|....|....|....|....|....|....|....|....|....|....|....|....|....|....|....|....|....|....|
AgoGspDN2      ---------------------------------------------------------------------------------------------------------------------------------- 1
RamGspDN2      ---------------------------------------------------------------------------------------------------------------------------------- 1
NgrGspDN2      ---------------------------------------------------------------------------------------------------------------------------------- 1
NfoGspDN2      ---------------------------------------------------------------------------------------------------------------------------------- 1
NdaGspDN2      ---------------------------------------------------------------------------------------------------------------------------------- 1
PcoWSGspDN2    ---------------------------------------------------------------------------------------------------------------------------------- 1
PcoAEGspDN2    ---------------------------------------------------------------------------------------------------------------------------------- 1
PkiGspDN2      ---------------------------------------------------------------------------------------------------------------------------------- 1
MjaGspDN2      ---------------------------------------------------------------------------------------------------------------------------------- 1
GokGspDN2      ---------------------------------------------------------------------------------------------------------------------------------- 1
SpiGspDN2      ---------------------------------------------------------------------------------------------------------------------------------- 1
E.coli         MFWR--------DMT----------------------------------------------LSIWRKKTTGLKTKKR-LLPLMLAA-----------------------ALCSSPVWAEEATFTANFKDT 52
V.cholerae     -------------------------------------------------------------MKYWLKKSSWL-----------LAG-----------------------SLLSTPL-AMANEFSASFKGT 34
A.salmonicida  ---------------------------------------------------------------MINKGKSWR-------LAT-VAA-----------------------ALMMAGS-AWATEYSASFKNA 35
P.aeruginosa   MSQP--------LLR----------------------------------------------ALFAPSSRSYVPAVLL-SLALGIQAAH--------------AENSGGNAFVPAGN-QQEAHWTINLKDA 60
N.meningtitis  MNTK--------LTKIISGLFVATAAFQTASAGNITDIKVSSLPNKQKIVKVSFDKEIVNPTGFVTSSPARIALDF---EQTGISMDQQVLEYADPLLSKISAAQNSSRARLVLNL-NKPGQYNTEVRGN 118
M.xanthus      MLEESAVTRGKWMLAAAWAVVLVGARVHGAELNTLRGLDVSRTGSGAQVVVTGTRPPTF--TVFRLSGPERLVVDLSSADATGIKGHHEGSGPVSGVVASQFSDQRASVGRVLLAL-DKASQYDVRADGN 127
S.enterica     LYNKNYPLRGD----------------------------------------------------------------------------------------------------------NRKGTF------- 17

                      140       150       160       170       180       190       200       210       220       230       240       250       260
               ....|....|....|....|....|....|....|....|....|....|....|....|....|....|....|....|....|....|....|....|....|....|....|....|....|....|
AgoGspDN2      ---------------------------------------------------------------------------------------------------------------------------------- 1
RamGspDN2      ---------------------------------------------------------------------------------------------------------------------------------- 1
NgrGspDN2      ---------------------------------------------------------------------------------------------------------------------------------- 1
NfoGspDN2      ---------------------------------------------------------------------------------------------------------------------------------- 1
NdaGspDN2      ---------------------------------------------------------------------------------------------------------------------------------- 1
PcoWSGspDN2    ---------------------------------------------------------------------------------------------------------------------------------- 1
PcoAEGspDN2    ---------------------------------------------------------------------------------------------------------------------------------- 1
PkiGspDN2      ---------------------------------------------------------------------------------------------------------------------------------- 1
MjaGspDN2      ---------------------------------------------------------------------------------------------------------------------------------- 1
GokGspDN2      ---------------------------------------------------------------------------------------------------------------------------------- 1
SpiGspDN2      ---------------------------------------------------------------------------------------------------------------------------------- 1
E.coli         DLKSFIETVGANLNK--------------TIIMGPG----------VQGKVSIRTMTPLNER------------------QYYQLFLNLLEAQG----------------------YAV----------- 107
V.cholerae     DIQEFINIVGRNLEK--------------TIIVDPS----------VRGKVDVRSFDTLNEE------------------QYYSFFLSVLEVYG----------------------FAV----------- 89
A.salmonicida  DIEEFINTVGKNLSK--------------TIIIEPS----------VRGKINVRSYDLLNEE------------------QYYQFFLSVLDVYG----------------------FAV----------- 90
P.aeruginosa   DIREFIDQISEITGE--------------TFVVDPR----------VKGQVSVVSKAQLSLS------------------EVYQLFLSVMSTHG----------------------FTV----------- 115
N.meningtitis  KVWIFINESDDTVSA-----PAR-----PAVKAAPAA-------PAKQQAAAPSTKSAVSVSE---------PFTPAKQQAAAPFTESVVSVSAP---------------------FSP----------- 190
M.xanthus      RVVISVDGAAQSVEAKRAEPPARTEGVTASVEVKPHSVSAAAPAKVVQAESAAVSKAALPENVVAAEADEREVSNPAQHITAMSFADDTLSIRADGDIARYEVLELADPPRLAVDLFGVGLATRAPRVKS 257
S.enterica     ---------------------------------------------------------------------------------------------------------------------------------- 17

                      270       280       290       300       310       320       330       340       350       360       370       380       390
               ....|....|....|....|....|....|....|....|....|....|....|....|....|....|....|....|....|....|....|....|....|....|....|....|....|....|
AgoGspDN2      ---------------------------------------------------------------------------------------------------------------------------------- 1
RamGspDN2      ---------------------------------------------------------------------------------------------------------------------------------- 1
NgrGspDN2      ---------------------------------------------------------------------------------------------------------------------------------- 1
NfoGspDN2      ---------------------------------------------------------------------------------------------------------------------------------- 1
NdaGspDN2      ---------------------------------------------------------------------------------------------------------------------------------- 1
PcoWSGspDN2    ---------------------------------------------------------------------------------------------------------------------------------- 1
PcoAEGspDN2    ---------------------------------------------------------------------------------------------------------------------------------- 1
PkiGspDN2      ---------------------------------------------------------------------------------------------------------------------------------- 1
MjaGspDN2      ---------------------------------------------------------------------------------------------------------------------------------- 1
GokGspDN2      ---------------------------------------------------------------------------------------------------------------------------------- 1
SpiGspDN2      ---------------------------------------------------------------------------------------------------------------------------------- 1
E.coli         -----------------------------VPMENDVLKVV----------------------------KSSAAKVEPLPLVG----EGSDNYAGDEMVTKV----------------------------- 147
V.cholerae     -----------------------------VEMDNGVLKVI----------------------------KSKDAKTSAIPVL-----SGEERANGDEVITQV----------------------------- 128
A.salmonicida  -----------------------------VPMDNGVLKVV----------------------------RSKDAKTSAIPVV-----DETNPGIGDEMVTRV----------------------------- 129
P.aeruginosa   -----------------------------VAQGDQA-RIV----------------------------PNAEAKTEA----------GGGQSAPDRLETRV----------------------------- 148
N.meningtitis  ----------------------------------------A---KQQAAASAKQQ-------------AAAPAKQQAAAPAKQTNIDFRKDGKNAGIIELAALGFAGQPDISQQHDHIIVTLKNHTLPTT 264
M.xanthus      GALRDVRVGAHADKVRLVLDVRGTMPAYRVDRANRGLEVVLGRAVARKAAPAVETQAVVASVAEVEPLRQAPVKSEASPVVEVKDVRFEESSAGGRIVMKLSGTSAWKVDRPDPRSAVL-TLENARLPKK 386
S.enterica     ---------------------------------------------------------------------------------------------------------------------------------- 17

                      400       410       420       430       440       450       460       470       480       490       500       510       520
               ....|....|....|....|....|....|....|....|....|....|....|....|....|....|....|....|....|....|....|....|....|....|....|....|....|....|
AgoGspDN2      ---------------------------------------------------------------------------------------------------------------------------------- 1
RamGspDN2      ---------------------------------------------------------------------------------------------------------------------------------- 1
NgrGspDN2      ---------------------------------------------------------------------------------------------------------------------------------- 1
NfoGspDN2      ---------------------------------------------------------------------------------------------------------------------------------- 1
NdaGspDN2      ---------------------------------------------------------------------------------------------------------------------------------- 1
PcoWSGspDN2    ---------------------------------------------------------------------------------------------------------------------------------- 1
PcoAEGspDN2    ---------------------------------------------------------------------------------------------------------------------------------- 1
PkiGspDN2      ---------------------------------------------------------------------------------------------------------------------------------- 1
MjaGspDN2      ---------------------------------------------------------------------------------------------------------------------------------- 1
GokGspDN2      ---------------------------------------------------------------------------------------------------------------------------------- 1
SpiGspDN2      ---------------------------------------------------------------------------------------------------------------------------------- 1
E.coli         -----------VPVRNVSVRELAPI---LRQMIDSAGSGN-VVNYDP-----------------------------------------------SNVIMLTGRASVVERLTEVIQRV---------DHAG 206
V.cholerae     -----------VAVKNVSVRELSPL---LRQLIDNAGAGN-VVHYDP-----------------------------------------------ANIILITGRAAVVNRLAEIIRRV---------DQAG 187
A.salmonicida  -----------VPVRNVSVRELAPL---LRQLNDNAGGGN-VVHYDP-----------------------------------------------SNVLLITGRAAVVNRLVEVVRRV---------DKAG 188
P.aeruginosa   -----------IQVQQSPVSELIPL---IRPLVPQ--YGH-LAAVPS-----------------------------------------------ANALIISDRSANIARIEDVIRQL---------DQKG 205
N.meningtitis  LQRSLDVADFKTPVQKVTLKRLNND---TQLIITTAGNWE-LVNKSAAPGYFTFQVLPKKQNLESGGVNNAPKTF------------------TGRKISLDFQDVEIRTILQILAKESGMNIVASDSVNG 372
M.xanthus      FERSLDTSALDTPVKMISAFSVPGEGGKVRLVVAADGAIEEKVSQSA--GTLSWRLDVKGVKTEEVAVAQRTAGFTTEAPAYAAEGAPQQARYRGKRVSFEFKDIDIQNLLRVIAEISKKNIVVADDVSG 514
S.enterica     -----------------------------------------YVSGPP-----------------------------------------------VYVDMVVNAATMMDKQNDGIE-------------LG 46

                      530       540       550       560       570       580       590       600       610       620       630       640       650
               ....|....|....|....|....|....|....|....|....|....|....|....|....|....|....|....|....|....|....|....|....|....|....|....|....|....|
AgoGspDN2      --------------------------------------------------------------------------------------------------MTSNGSSSGGHEALSTPLPLLQSSVKAYVPPS 32
RamGspDN2      ----------------------------------------------------------------------------------------------------------------MDDGERFRSTVFSQRAPA 18
NgrGspDN2      ------------------------------------------MQESKQTNNGSVLNRQQDNKNEKQFLSETNTHETDNDKQINN-------KIEDGEQNMNTTTTAQPTLNNYQ-QVHHRSQVKTYTPPV 80
NfoGspDN2      ---------------------------------------------------------------------------------------------------MNNSS--------------LRSQVKTYTPPP 17
NdaGspDN2      ---------------------------------------------------------------------------------------------------MNKK------EETPKTSVDEKSTVKTYRPP- 24
PcoWSGspDN2    ----------------------------------------------------------------------------------------MSPSQPAAPTKPHANRRSKNPRDNKPKNTSMKSSVKTFRPPP 42
PcoAEGspDN2    ------------------------------------------------------------------------------------------------------------------------STVKTYRPP- 9
PkiGspDN2      ------------------------------------------MPSSKEENESQPSPQSPPHEEHKNPSLPRKKSKRSSSKQKKNRSSKQKQKQQEQKQKQNQKQQKQPPETTHRPSSSTKSTVKTYRPP- 87
MjaGspDN2      -----------------------------------------------------------------------------------------------------------------------MSTVSSFRPPE 11
GokGspDN2      ----------------------------------------------------------------------------------------------------------MVDPPPAAAIDDNGSSVACYRTPQ 24
SpiGspDN2      ---------------------------------------------------------------------------------------------------------------------------------- 1
E.coli         NRTEEVIPLDNASASEIARVLESLTKNS---GENQPATLKSQIVADERTNSVIVSGDPATRDKMRRLIRRLDSEMERSGNS----------QVFYLKYSKAEDLVDVLKQVSGTLTAAKEEAEGTVGSGR 323
V.cholerae     DKEIEVVELNNASAAEMVRIVEALNKTTD--AQNTPEFLKPKFVADERTNSILISGDPKVRERLKRLIKQLDVEMAAKGNN----------RVVYLKYAKAEDLVEVLKGVSENLQAEKGTGQPTT-SKR 304
A.salmonicida  DQEVDIIKLKYASAGEMVRLVTNLNKDGNTQGGNTSLLLAPKVVADERTNSVVVSGEPKARARIIQMVRQLDRDLQSQGNT----------RVFYLKYGKAKDMVEVLKGVSTSIEADKKGGGATAGGGN 308
P.aeruginosa   SHDYSVINLRYGWVMDAAEVLNNAMSRGQAKGA-----AGAQVIADARTNRLIILGPPQARAKLVQLAQSLDTPTARSANT----------RVIRLRHNDAKTLAETLGQISEGMKNNGGQGGEQTGGGR 320
N.meningtitis  KMTLSLKDVPWDQALDLV--------------------MQARNLDMRQQGNIVNIAP------RDELLAKDKAFLQAEKDIA-D-LGALYSQNFQLKYKNVEEFRSILRLDNADTTGNRN----TLVSGR 470
M.xanthus      KVTIRLRNVPWDQALDLV--------------------LRTKALGKEEFGNIIRIAPLKTLEEEARLRQERKASLQRQEDLMV--------NLIPVNYAVAADMAARVK---------------DVLSER 601
S.enterica     RQKIGVMRLNNTFVGDRT--------------------------YNLRDQKMVIPGIATAIERLLQGEEQPLGNIVSSEPPAMPAFSANGEKGKAANYAGGMSLQEALKQ--------------NAAAGN 136

                      660       670       680       690       700       710       720       730       740       750       760       770       780
               ....|....|....|....|....|....|....|....|....|....|....|....|....|....|....|....|....|....|....|....|....|....|....|....|....|....|
AgoGspDN2      MTDGG----SGGDEPGRPLLCIHTRPSYMKRISEVLLRLDTPPQMLQLEFQLVEVAEDDLTQLRHRINHPVLNRIAGESAMQDDVFSRRAYSPMDIRAANTSGKCNVLQPQMQVQSRL------------ 146
RamGspDN2      ELAGE--SGGGGSGQAAAVVCVHTYPHLMRRLVAFLRHFDKPPKNLSLEFTFVEVADEDVSQFRYHLQHPLLRSALRAPAPP-E----------MRQPPEPRGLPSQLSANL------------------ 117
NgrGspDN2      LGSP---SGSSGGIGTTPVVSIYTKPHLMKRLKQFLDHFDRAPQQMFIDFAFVEVEENDVSQFRYKLQHPILQSIYGPTVDEQL------PLYQNESTSSSARLPSSLTPRL------------------ 183
NfoGspDN2      MSDTPHSSPSSSVASTTPVVSIYTKPHLMRRLKQFLDHFDRPPQQLFIDFAFVEVEENDVSQFRYKLQHPILQSIYGPTVDEQL------PLYQNESTSSSARLPTSLTPRL------------------ 123
NdaGspDN2      -------------DGSTPVVCIHTKPSLMKKLMSFLQHFDRPPQQLFLEFVFVEVEENDAAQFRYRLNHPMLNSITGGDIDSDLLNMQ--PFQQTS-NQPSTRLPSELNPRM------------------ 120
PcoWSGspDN2    GDDG--------ATLQSSMLLIHTYPSLHQRLLQFLSHFDKPPQQMYFELLFTEMRDVDVKQMRWKFGDNVLSG-----AQMFD------QMVNGMNQMENVRLPQELQGKM------------------ 135
PcoAEGspDN2    --DG-----------SKPMICIHTKPTLHKRLLKFLEHFDKPPQQLYLEFSFVEVAEDDVAQFRYKLDHPVLNEIMG-TDGVYS------SMIN-AESTP-THLPSSLQPKM------------------ 99
PkiGspDN2      -------------DGSPPVLCIHTRPHLMKKLRAFFEHFDRPPQQLFLEFVFVEVAEEDAAQFRYRLEHPVIDSVYGRAADADV------PLSQRE-RRRHPELPDELLPRL------------------ 179
MjaGspDN2      ----------GGQ----PVVAIHTTPALMKKLVAFLKHFDRPPQQVYLEFVFLEVAEDDLAEFRYNVQHPLLYRLLGR-APP------------VNAPMAGSDAAAAPPPSL------------------ 96
GokGspDN2      ----------GGQ----PVIAVHTTPALMRKLVAFLRHFDRPPQQLFLEFVFLEVSEDELNEFRYNLQHPLLYRLLGR-TPP------------ATAPAPSAASTSTAAPV------------------- 108
SpiGspDN2      ----------GGQ----PVVCIHTTPALMARLTEFLRHFDRPPQQLFLEFLFVEMASEELEQLRYHLRHPLLSQMLGAPVPPSA----------VTETP----LPEQEP--------------------- 81
E.coli         -EVV-----SIAASKHSNALIVTAPQDIMQSLQSVIEQLDIRRAQVHVEALIVEVAEGSNINFGVQWGSK-----DAG----LMQFANGTQIPIGTLGAAISAAKPQKGSTVISENGATTINPDTN---G 435
V.cholerae     -NEV-----MIAAHADTNSLVLTAPQDIMNAMLEVIGQLDIRRAQVLIEALIVEMAEGDGINLGVQWGSL-----ESG---SVIQYGN-TGASIGNVMIGLEEAKDTTQTKAVYDTNNNFLRNETTTTKG 419
A.salmonicida  -ASIGGGKLAISADETTNALVITAQPDVMAELEQVVAKLDIRRAQVLVEAIIVEIADGDGLNLGVQWANT-----NGG----GTQFTN-AGPGIGSVAIAAKDYKDN----------------------G 405
P.aeruginosa   PSNI-----LIRADESTNALVLLADPDTVNALEDIVRQLDVPRAQVLVEAAIVEISGDIQDAVGVQWAIN-----KGGMGGTKTNFAN-TGLSIGTLLQSLESNKAP----------------------- 416
N.meningtitis  ------G--SVLIDPATNTLIVTDTRSVIEKFRKLIDELDVPAQQVMIEARIVEAADGFSRDLGVKFG-------ATG----KKKLKN-DTSAFGWGVNSGFGGDDK--------WGAETKINLPI---- 568
M.xanthus      ------G--SVTVDQRTNVLIVKDVRSNTERARSLVRSLDTQTPQVLIESRIVEANTSFSRSLGVQWGGQARAGQATG-NSTGLIFPN-NLAVTGGVTGTGAGLPDN----------PNFAVNLPT---G 708
S.enterica     ---I-----KIVAYPDTNSLLVKGTAEQVHFIEMLVKALDVAKRHVELSLWIVDLNKSDLERLGTSWSGSITIGDKLG---------------------------------------------------- 206
```

```
                  790       800       810       820       830       840       850       860       870       880       890       900       910
          ....|....|....|....|....|....|....|....|....|....|....|....|....|....|....|....|....|....|....|....|....|....|....|....|....|....|
AgoGspDN2      ---------------------------------------------------------------------------------------------------------------------------------- 146
RamGspDN2      ---------------------------------------------------------------------------------------------------------------------------------- 117
NgrGspDN2      ---------------------------------------------------------------------------------------------------------------------------------- 183
NfoGspDN2      ---------------------------------------------------------------------------------------------------------------------------------- 123
NdaGspDN2      ---------------------------------------------------------------------------------------------------------------------------------- 120
PcoWSGspDN2    ---------------------------------------------------------------------------------------------------------------------------------- 135
PcoAEGspDN2    ---------------------------------------------------------------------------------------------------------------------------------- 99
PkiGspDN2      ---------------------------------------------------------------------------------------------------------------------------------- 179
MjaGspDN2      ---------------------------------------------------------------------------------------------------------------------------------- 96
GokGspDN2      ---------------------------------------------------------------------------------------------------------------------------------- 108
SpiGspDN2      ---------------------------------------------------------------------------------------------------------------------------------- 81
E.coli         DLSTLAQLLSGFSGTAVGVVKGDWMALVQAVKNDSSSNVLSTPSITTLDNQEAFFMVGQDVPVLTGS--TVGSNNSNPFNTVERKKVGIMLKVTPQINEGNAVQMVIEQEV-SKV---EGQT-SLD--VV 556
V.cholerae     DYTKLASALSSIQGAAVSIAMGDWTALINAVSNDSSSNILSSPSITVMDNGEASFIVGEEVPVITGS--TAGSNNDNPFQTVDRKEVGIKLKVVPQINEGNSVQLNIEQEV-SNV---LGANGAVD--VR 541
A.salmonicida  TTTGIAKLAESFNGMAAGFYQGNWAMLVTALSTNTKSDILSTPSIVTMDNKEASFNVGQEVPVQTGK--QNSTSGDTTFSTIERKTVGTKLVVTPQINEGDSVLLTIEQEV-SSVGKQASGTEGLG--PT 530
P.aeruginosa   --------ESIPDGAIVGIGSSSFGALVTALSANTKSNLLSTPSLLTLDNQKAEILVGQNVPFQTGSYTTNSEGSSNPFTTVERKDIGVSLKVTPHINDGAALRLEIEQEI-SALLPNAQQRNNTD--LI 535
N.meningtitis  ----TAAANSISLVRAIS--SGALNLELSASESLSKTKTLANPRVLTQNRKEAKIESGYEIPFTVTSIANGGSSTNTEL-----KKAVLGLTVTPNITPDGQIIMTVKITK-DSPAQCASGN-QTI--LC 683
M.xanthus      TGQGVGGAMGFTFGSAGG--ALQLNLRLSAAENEGSVKTISAPKVTTLDNNTARISQGVSIPFSQTS----AQGVNTTF-----VEARLSLEVTPHITQDGSVLMSINASN-NQPDPSSTGA-NGQ--PS 823
S.enterica     --------VSLNQSSISTLDGSRFIAAVNALEEKKQATVVSRPVLLTQENVPAIFDNNRTFYTKLIG------ERNVAL---EHVTYGTMIRVLPRFSADGQIEMSLDIEDGNDKTPQSDTTTSVDALPE 319

                  920       930       940       950       960       970       980       990      1000      1010      1020      1030      1040
          ....|....|....|....|....|....|....|....|....|....|....|....|....|....|....|....|....|....|....|....|....|....|....|....|....|....|
AgoGspDN2      ---------------------------------------------------------------------------------------------------------------------------------- 146
RamGspDN2      ---------------------------------------------------------------------------------------------------------------------------------- 117
NgrGspDN2      ---------------------------------------------------------------------------------------------------------------------------------- 183
NfoGspDN2      ---------------------------------------------------------------------------------------------------------------------------------- 123
NdaGspDN2      ---------------------------------------------------------------------------------------------------------------------------------- 120
PcoWSGspDN2    ---------------------------------------------------------------------------------------------------------------------------------- 135
PcoAEGspDN2    ---------------------------------------------------------------------------------------------------------------------------------- 99
PkiGspDN2      ---------------------------------------------------------------------------------------------------------------------------------- 179
MjaGspDN2      ---------------------------------------------------------------------------------------------------------------------------------- 96
GokGspDN2      ---------------------------------------------------------------------------------------------------------------------------------- 108
SpiGspDN2      ---------------------------------------------------------------------------------------------------------------------------------- 81
E.coli         FGERKLKTTVLANDGELIVLGGLMDDQAGESVAKVPLLGDIPLIGNLFKSTADKKEKRNLMVFIRPTILRDGMAADGVSQRKYNYMRAEQIYRDEQ-GLSLMPHTAQPILP--AQNQALPPEVRAFLNAG 683
V.cholerae     FAKRQLNTSVMVQDGQMLVLGGLIDERALESESKVPLLGDIPLLGQLFRSTSSQVEKKNLMVFIKPTIIRDGVTADGITQRKYNYIRAEQLFRAEK-GLRLLDDASVPVLPKFGDDRRHSPEIQAFIEQM 670
A.salmonicida  FDTRTVKNAVLVKSGETVVLGGLMDEQTKEEVSKVPLLGDIPVLGYLFRSTSNTTSKRNLMVFIRPTILRDANVYSGISSNKYTLFRAQQLEAAAQKGYATSPDRQ--VLPEYGQDVVQSPEIQKQIELM 658
P.aeruginosa   TSKRSIKSTILAENGQVIVIGGLIQDDVSQAESKVPLLGDIPLLGRLFRSTKDTHTKRNLMVFLRPTVVRDSAGLAALSGKKYSDIRVIDGTRGPE-GRPS-------ILPTNANQLFDGQAVDLR-ELM 656
N.meningtitis  ISTKNLNTQAMVENGGTLIVGGIYEEDNGNTLTKVPLLGDIPVIGNLFKTRGKKTDRRELLIFITPRIMGTAG----------NSLRY------------------------------------------ 761
M.xanthus      IQRKEANTQVLVKDGDTTVIGGIYVRRGATQVNSVPFLSRIPVLGLLFKNNSETDTRQELLIFITPRILNRQTIAQTL---------------------------------------------------- 901
S.enterica     VGRTLISTIARVPHGKSLLVGGYTRDANTDTVQSIPFLGKLPLIGSLFRYSSKNKSNVVRVFMIEPKEIVDPLTPDA-SESVNNILKQSGAWSGDD---------------------KLQKWVRVYLDRG 427

                 1050
          ....|....|....|....
AgoGspDN2      ------------------- 146
RamGspDN2      ------------------- 117
NgrGspDN2      ------------------- 183
NfoGspDN2      ------------------- 123
NdaGspDN2      ------------------- 120
PcoWSGspDN2    ------------------- 135
PcoAEGspDN2    ------------------- 99
PkiGspDN2      ------------------- 179
MjaGspDN2      ------------------- 96
GokGspDN2      ------------------- 108
SpiGspDN2      ------------------- 81
E.coli         RTR---------------- 686
V.cholerae     EAKQ--------------- 674
A.salmonicida  KARQQATADGAQPFVQGNN 677
P.aeruginosa   TE----------------- 658
N.meningtitis  ------------------- 761
M.xanthus      ------------------- 901
S.enterica     QEAIK-------------- 432
```

(D) GspDN3

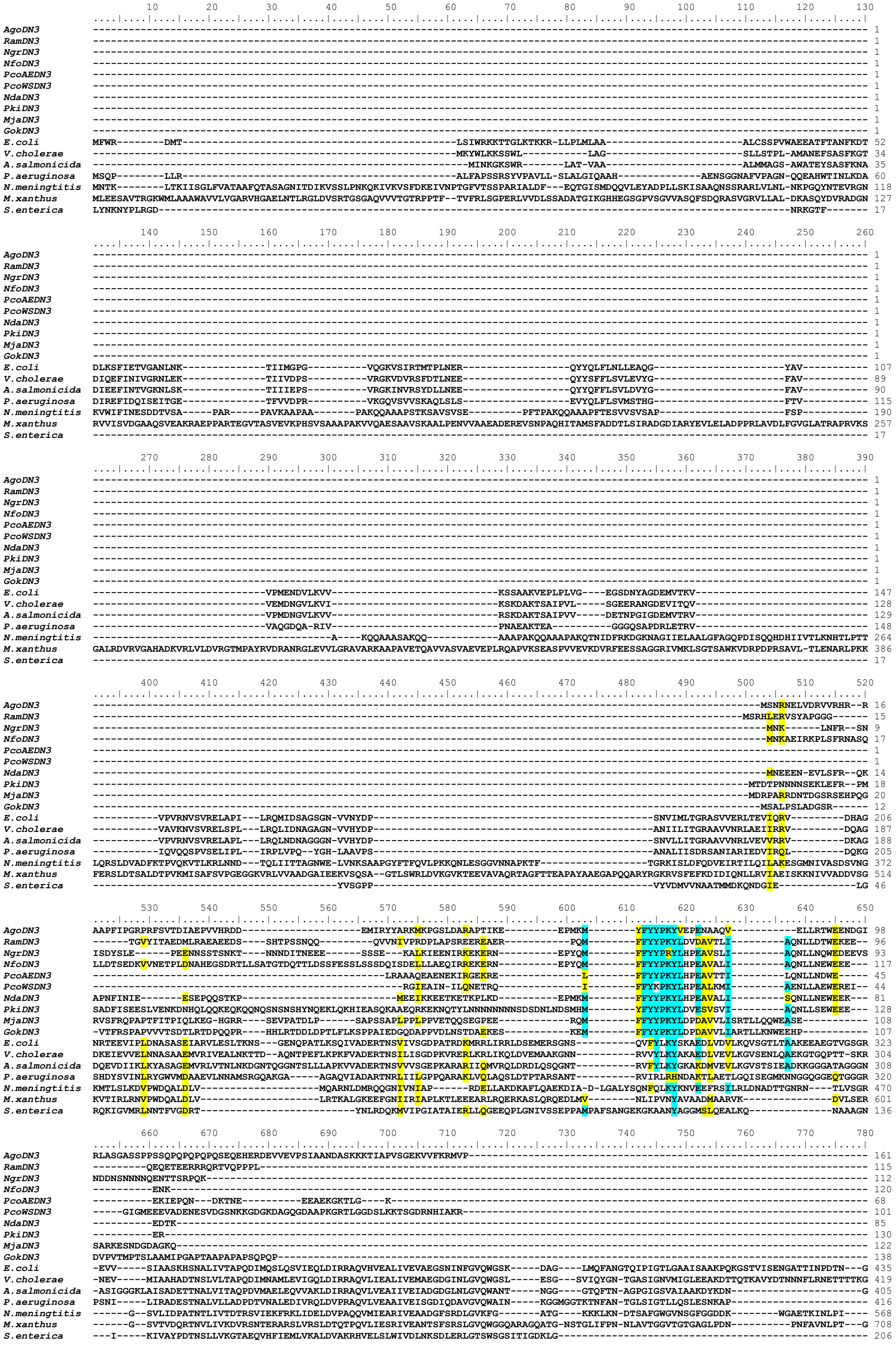

```
                        790       800       810       820       830       840       850       860       870       880       890       900       910
               ....|....|....|....|....|....|....|....|....|....|....|....|....|....|....|....|....|....|....|....|....|....|....|....|....|....|
AgoDN3         ---------------------------------------------------------------------------------------------------------------------------------- 161
RamDN3         ---------------------------------------------------------------------------------------------------------------------------------- 115
NgrDN3         ---------------------------------------------------------------------------------------------------------------------------------- 112
NfoDN3         ---------------------------------------------------------------------------------------------------------------------------------- 120
PcoAEDN3       ---------------------------------------------------------------------------------------------------------------------------------- 68
PcoWSDN3       ---------------------------------------------------------------------------------------------------------------------------------- 101
NdaDN3         ---------------------------------------------------------------------------------------------------------------------------------- 85
PkiDN3         ---------------------------------------------------------------------------------------------------------------------------------- 130
MjaDN3         ---------------------------------------------------------------------------------------------------------------------------------- 122
GokDN3         ---------------------------------------------------------------------------------------------------------------------------------- 138
E.coli         DLSTLAQLLSGFSGTAVGVVKGDWMALVQAVKNDSSSNVLSTPSITTLDNQEAFFMVGQDVPVLTGS--TVGSNNSNPFNTVERKKVGIMLKVTPQINEGNAVQMVIEQEV-SKV---EGQT-SLD--VV 556
V.cholerae     DYTKLASALSSIQGAAVSIAMGDWTALINAVSNDSSSNILSSPSITVMDNGEASFIVGEEVPVITGS--TAGSNNDNPFQTVDRKEVGIKLKVVPQINEGNSVQLNIEQEV-SNV---LGANGAVD--VR 541
A.salmonicida  TTTGIAKLAESFNGMAAGFYQGNWAMLVTALSTNTKSDILSTPSIVTMDNKEASFNVGQEVPVQTGK--QNSTSGDTTFSTIERKTVGTKLVVTPQINEGDSVLLTIEQEV-SSVGKQASGTEGLG--PT 530
P.aeruginosa   --------ESIPDGAIVGIGSSSFGALVTALSANTKSNLLSTPSLLTLDNQKAEILVGQNVPFQTGSYTTNSEGSSNPFTTVERKDIGVSLKVTPHINDGAALRLEIEQEI-SALLPNAQQRNNTD--LI 535
N.meningtitis  ----TAAANSISLVRAIS--SGALNLELSASESLSKTKTLANPRVLTQNRKEAKIESGYEIPFTVTSIANGGSSTNTEL-----KKAVLGLTVTPNITPDGQIIMTVKITK-DSPAQCASGN-QTI--LC 683
M.xanthus      TGQGVGGAMGFTFGSAGG--ALQLNLRLSAAENEGSVKTISAPKVTTLDNNTARISQGVSIPFSQTS----AQGVNTTF-----VEARLSLEVTPHITQDGSVLMSINASN-NQPDPSSTGA-NGQ--PS 823
S.enterica     --------VSLNQSSISTLDGSRFIAAVNALEEKKQATVVSRPVLLTQENVPAIFDNNRTFYTKLIG------ERNVAL---EHVTYGTMIRVLPRFSADGQIEMSLDIEDGNDKTPQSDTTTSVDALPE 319

                        920       930       940       950       960       970       980       990      1000      1010      1020      1030      1040
               ....|....|....|....|....|....|....|....|....|....|....|....|....|....|....|....|....|....|....|....|....|....|....|....|....|....|
AgoDN3         ---------------------------------------------------------------------------------------------------------------------------------- 161
RamDN3         ---------------------------------------------------------------------------------------------------------------------------------- 115
NgrDN3         ---------------------------------------------------------------------------------------------------------------------------------- 112
NfoDN3         ---------------------------------------------------------------------------------------------------------------------------------- 120
PcoAEDN3       ---------------------------------------------------------------------------------------------------------------------------------- 68
PcoWSDN3       ---------------------------------------------------------------------------------------------------------------------------------- 101
NdaDN3         ---------------------------------------------------------------------------------------------------------------------------------- 85
PkiDN3         ---------------------------------------------------------------------------------------------------------------------------------- 130
MjaDN3         ---------------------------------------------------------------------------------------------------------------------------------- 122
GokDN3         ---------------------------------------------------------------------------------------------------------------------------------- 138
E.coli         FGERKLKTTVLANDGELIVLGGLMDDQAGESVAKVPLLGDIPLIGNLFKSTADKKEKRNLMVFIRPTILRDGMAADGVSQRKYNYMRAEQIYRDEQ-GLSLMPHTAQPILP--AQNQALPPEVRAFLNAG 683
V.cholerae     FAKRQLNTSVMVQDGQMLVLGGLIDERALESESKVPLLGDIPLLGQLFRSTSSQVEKKNLMVFIKPTIIRDGVTADGITQRKYNYIRAEQLFRAEK-GLRLLDDASVPVLPKFGDDRRHSPEIQAFIEQM 670
A.salmonicida  FDTRTVKNAVLVKSGETVVLGGLMDEQTKEEVSKVPLLGDIPVLGYLFRSTSNTTSKRNLMVFIRPTILRDANVYSGISSNKYTLFRAQQLEAAAQKGYATSPDRQ--VLPEYGQDVVQSPEIQKQIELM 658
P.aeruginosa   TSKRSIKSTILAENGQVIVIGGLIQDDVSQAESKVPLLGDIPLLGRLFRSTKDTHTKRNLMVFLRPTVVRDSAGLAALSGKKYSDIRVIDGTRGPE-GRPS-------ILPTNANQLFDGQAVDLR-ELM 656
N.meningtitis  ISTKNLNTQAMVENGGTLIVGGIYEEDNGNTLTKVPLLGDIPVIGNLFKTRGKKTDRRELLIFITPRIMGTAG----------NSLRY------------------------------------------ 761
M.xanthus      IQRKEANTQVLVKDGDTTVIGGIYVRRGATQVNSVPFLSRIPVLGLLFKNNSETDTRQELLIFITPRILNRQTIAQTL---------------------------------------------------- 901
S.enterica     VGRTLISTIARVPHGKSLLVGGYTRDANTDTVQSIPFLGKLPLIGSLFRYSSKNKSNVVRVFMIEPKEIVDPLTPDA-SESVNNILKQSGAWSGDD---------------------KLQKWVRVYLDRG 427

                       1050
               ....|....|....|....
AgoDN3         ------------------- 161
RamDN3         ------------------- 115
NgrDN3         ------------------- 112
NfoDN3         ------------------- 120
PcoAEDN3       ------------------- 68
PcoWSDN3       ------------------- 101
NdaDN3         ------------------- 85
PkiDN3         ------------------- 130
MjaDN3         ------------------- 122
GokDN3         ------------------- 138
E.coli         RTR---------------- 686
V.cholerae     EAKQ--------------- 674
A.salmonicida  KARQQATADGAQPFVQGNN 677
P.aeruginosa   TE----------------- 658
N.meningtitis  ------------------- 761
M.xanthus      ------------------- 901
S.enterica     QEAIK-------------- 432
```

Supplementary Fig. 4A-D Multiple sequence alignments of eukaryotic GspD, GspDN1, GspDN2 and GspDN3 with bacterial GspD proteins. For further details see page 23.

(E) GspE

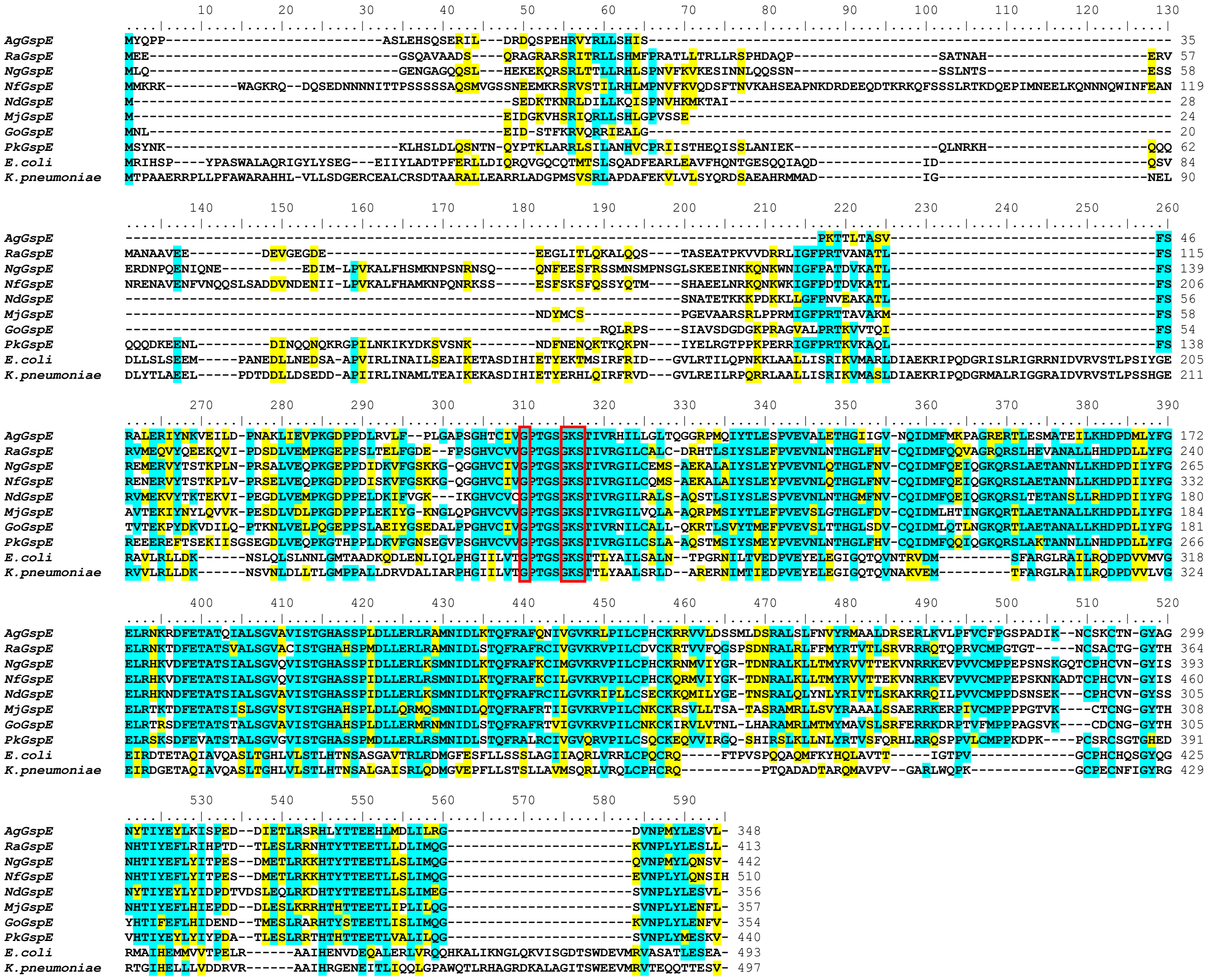
AgGspE
RaGspE
NgGspE
NfGspE
NdGspE
MjGspE
GoGspE
PkGspE
E.coli
K.pneumoniae

(F) GspEL

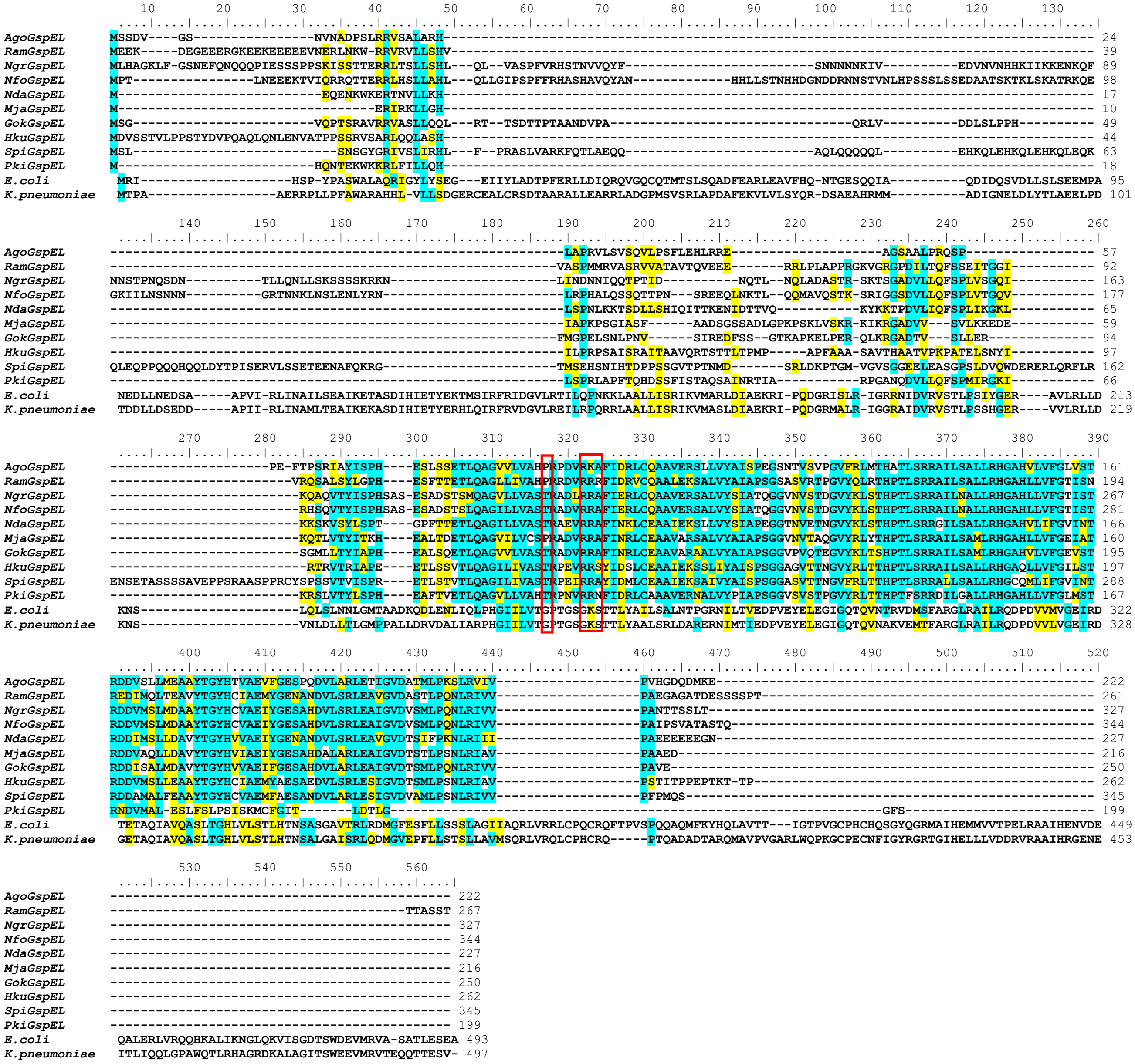
AgoGspEL
RamGspEL
NgrGspEL
NfoGspEL
NdaGspEL
MjaGspEL
GokGspEL
HkuGspEL
SpiGspEL
PkiGspEL
E.coli
K.pneumoniae

(G) GspEN2A

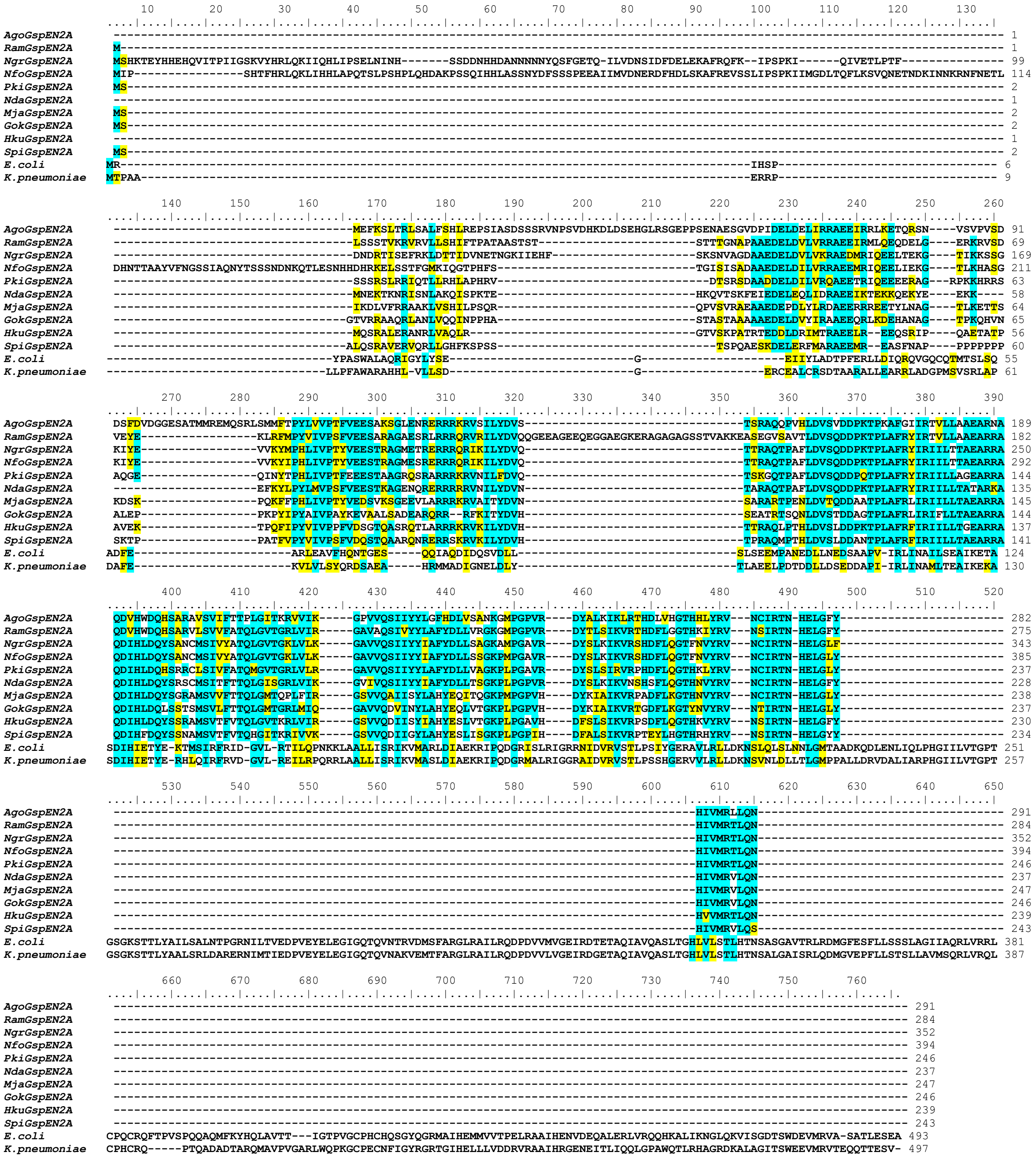

(H) GspEN2B

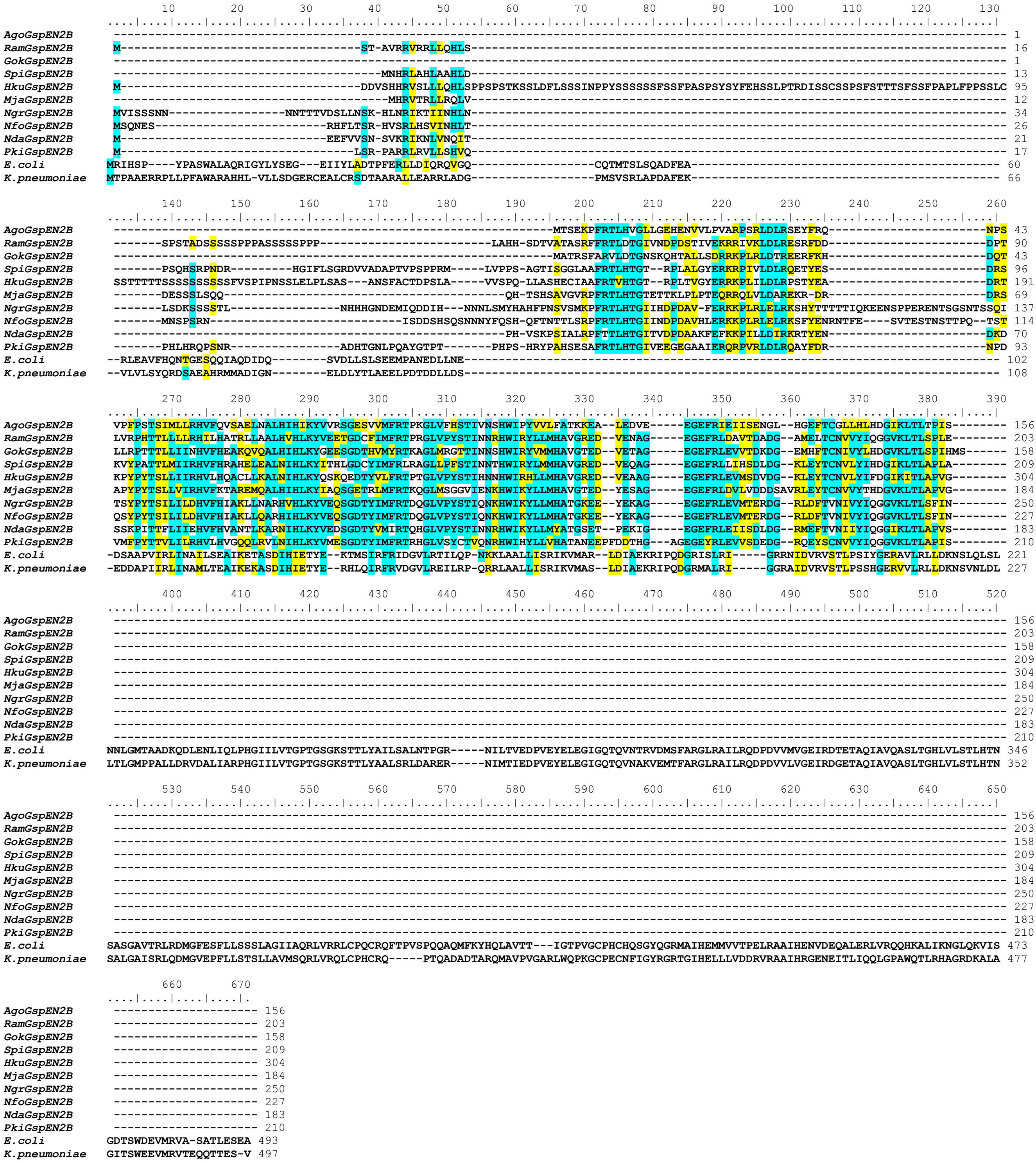

Supplementary Fig. 4E-H Protein sequence alignments of eukaryotic GspE, GspEL, GspEN2A and GspEN2B with reference bacterial GspE proteins. For further details see page 23.

(I) GspF

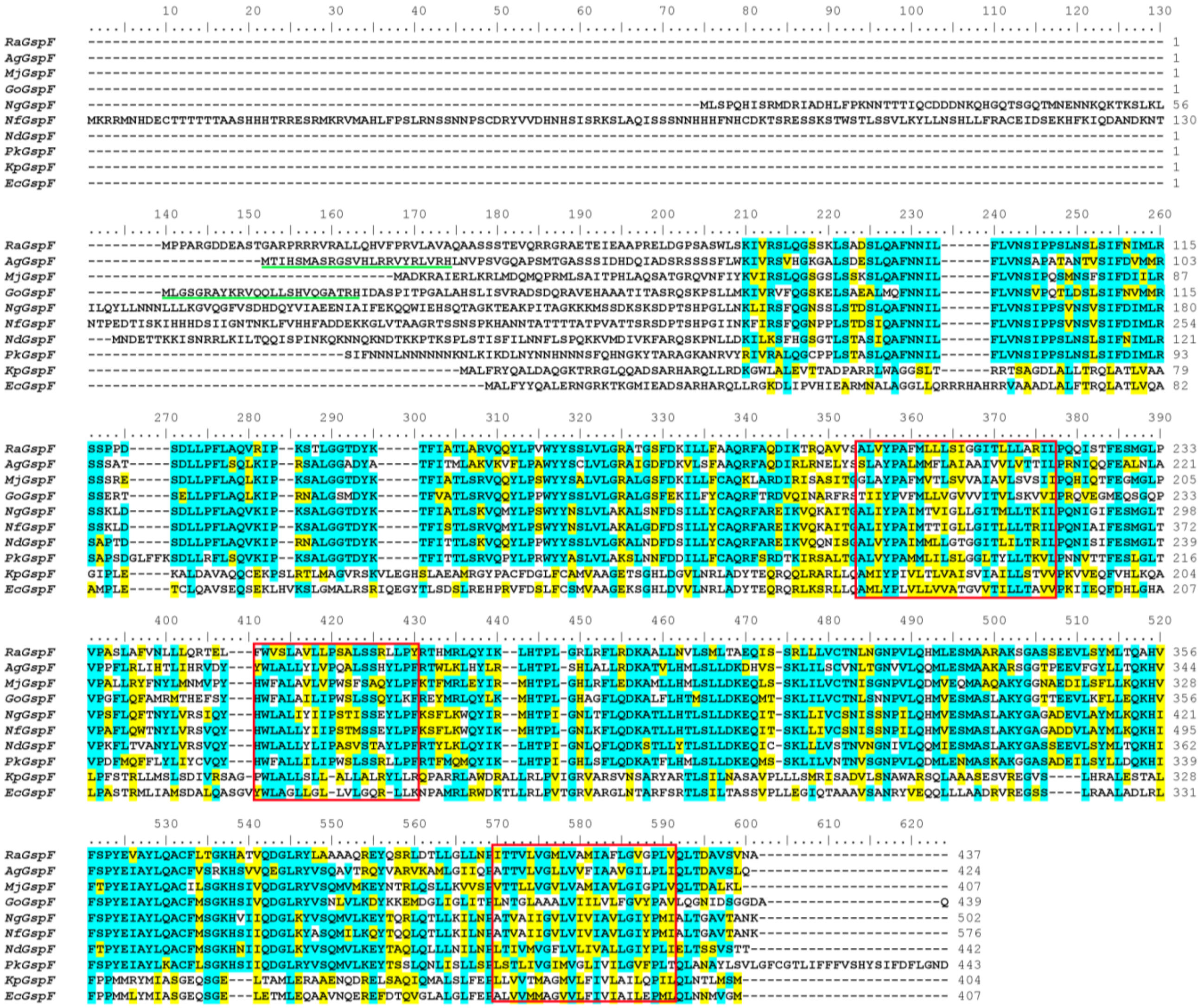

Supplementary Fig. 4I Protein sequence alignments of eukaryotic and reference bacterial GspF proteins. For further details see page 23.

(J) GspG

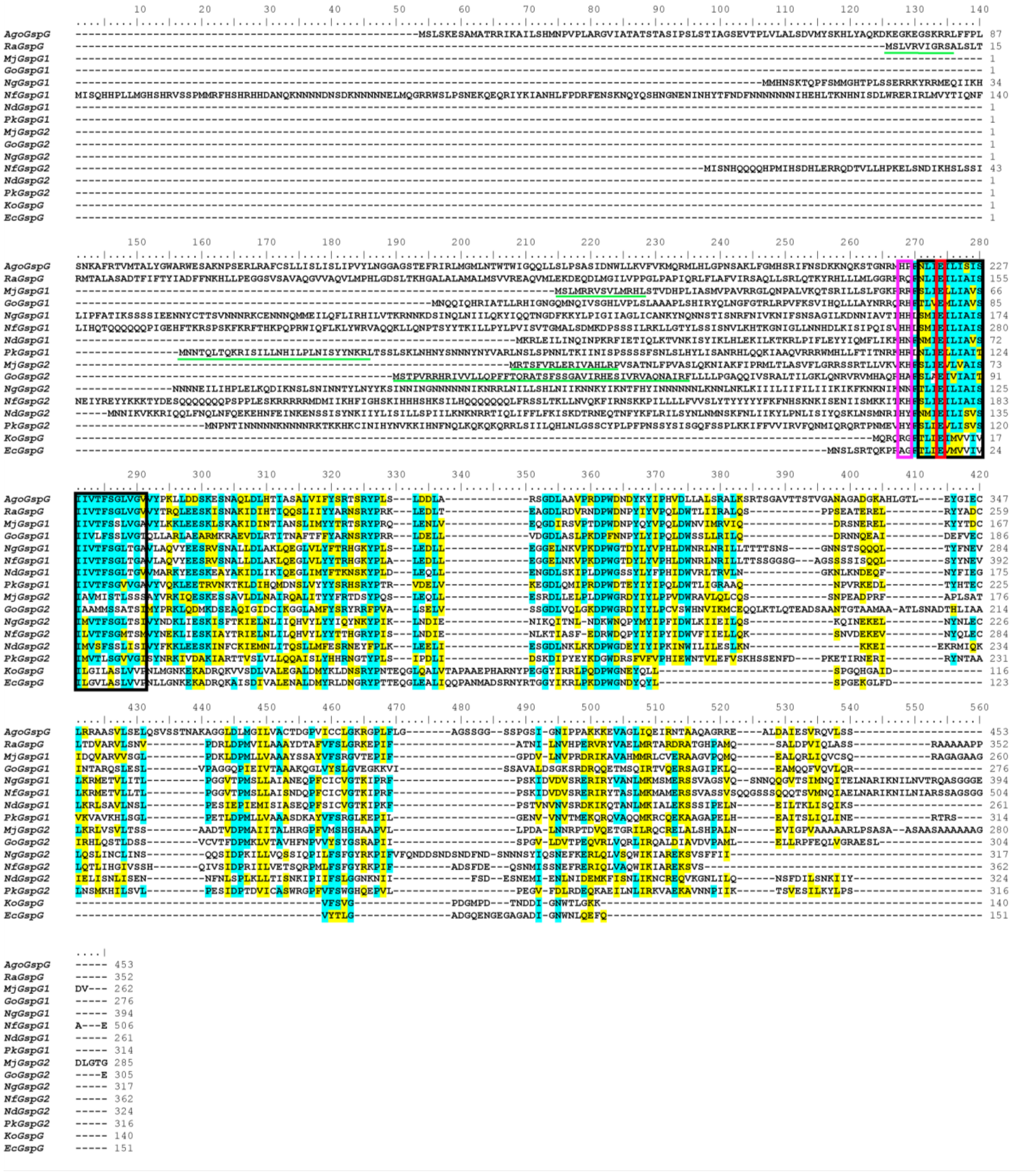

Supplementary Fig. 4J Protein sequence alignments of eukaryotic and reference bacterial GspG proteins. For further details see page 23.

Supplementary Fig. 4 (pg. 8-22) Multiple sequence alignments of eukaryotic GspD (panel A-D, pg. 8-16), GspE (panel E-H, pg. 17-20), GspF (panel I, pg. 21) and GspG (panel J, pg. 22) proteins with their prokaryotic homologues. The alignments illustrate the presence of N-terminal sequence extensions in the eukaryotic proteins, with targeting sequences recognized by MitoFates predictor[2] underlined in green. Identical and similar residues are highlighted in turquoise and yellow, respectively (using 50% conservation of the position as the threshold). **A.** In addition to the initially identified eukaryotic GspD proteins, representative sequences of GspDL, a more divergent paralogue identified by phylogenetic profiling, are included in the alignment together with reference bacterial GspD sequences. **B-D.** Three eukaryotic proteins homologous to the N-domain of bacterial secretins. While GspDN1 corresponds to a full single N-domain, GspDN2 and GspDN3 relate to its C-terminal and the N-terminal halves.
E-H. In addition to GspE proteins, three additional eukaryotic proteins GspEL, GspEN2A and GspEN2B were identified. While the bacterial GspE proteins contain four domains referred to as N1E, N2E, C1E and C2E, all four eukaryotic GspE proteins (GspE, GspEL, GspN2A and GspN2B) contain different domain variants. GspE proteins (**E**) carry C1E and C2E domains, GspEL proteins (**F**) contain C1E domain and GspEN2A and GspEN2B (**G** and **H**) contain N-domains. Only eukaryotic GspE proteins carry GxxxGK[ST] motif (also called the Walker A motif or the P-loop) functionally critical for the ATPase function of the protein. **I.** Transmembrane domains of GspF are highlighted by red rectangles. **J.** A conserved polar anchor of GspG is highlighted by the magenta rectangle and the transmembrane domain by a black rectangle, while the absolutely conserved glutamic acid residue at the +5 position relative to the pseudopilin processing site is highlighted in red.
Species abbreviations: *Mj – Malawimonas jakobiformis*, *Go – Gefionella okellyi*, *Ra – Reclinomonas americana*, *Ag – Andalucia godoyi*, *Nd – Neovahlkampfia damariscottae*, *Pk – Pharyngomonas kirbyi*, *Ng – Naegleria gruberi*, *Nf – Naegleria fowleri*, *Ko - Klebsiella oxytoca, Kp – Klebsiella pneumoniae, Ec – Escherichia coli.*

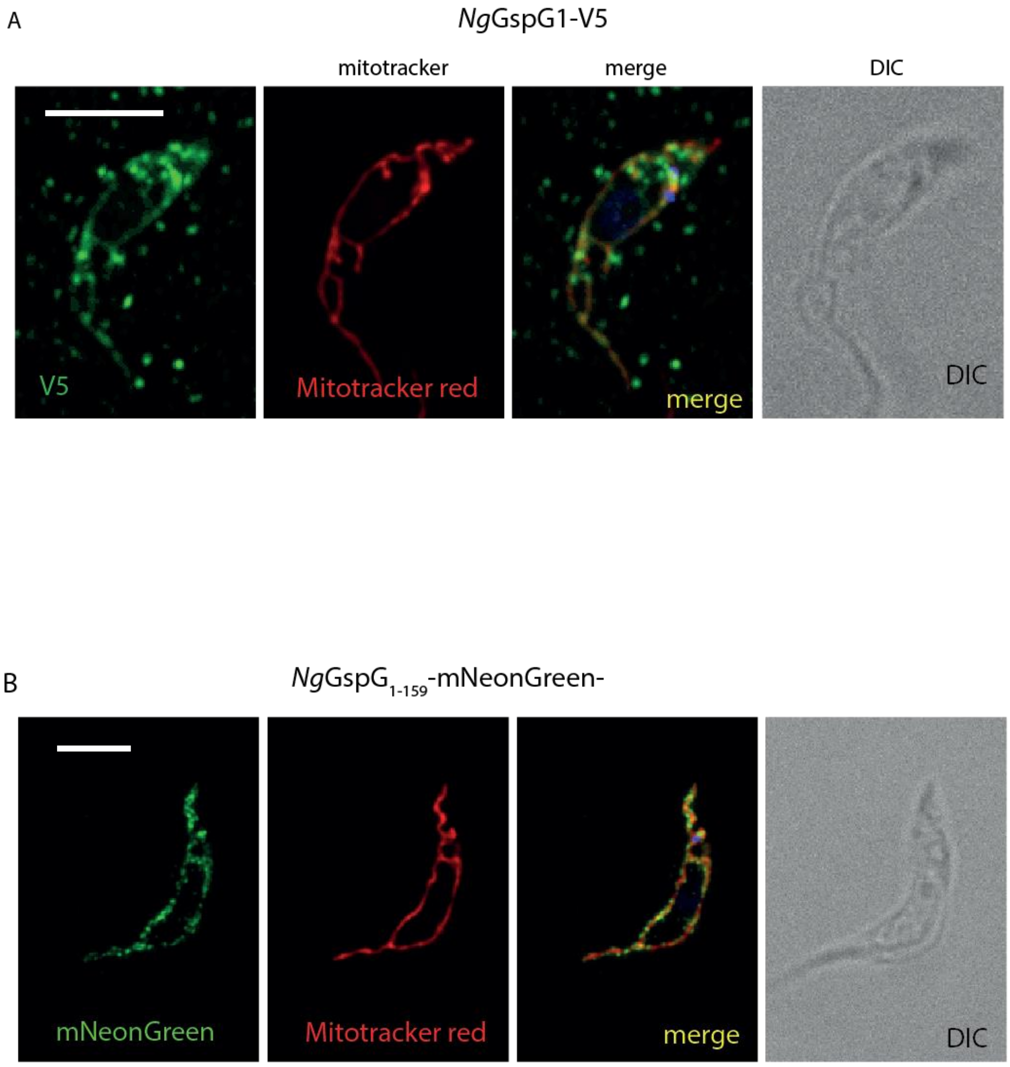

Supplementary Figure 5. *Ng*GspG1 expressed in *T. brucei.* The expression of *Ng*GspG1 (A) and the N-terminal part of the protein (residues 1-159) fused with mNeonGreen (B) shows the mitochondrial localization of the constructs. In the case of full-length *Ng*GspG1 with the C-terminal V5 tag, only very weak expression upon high contrasting could be detected. (representative images of three experiments are shown). Scale bar 10 µm.

A

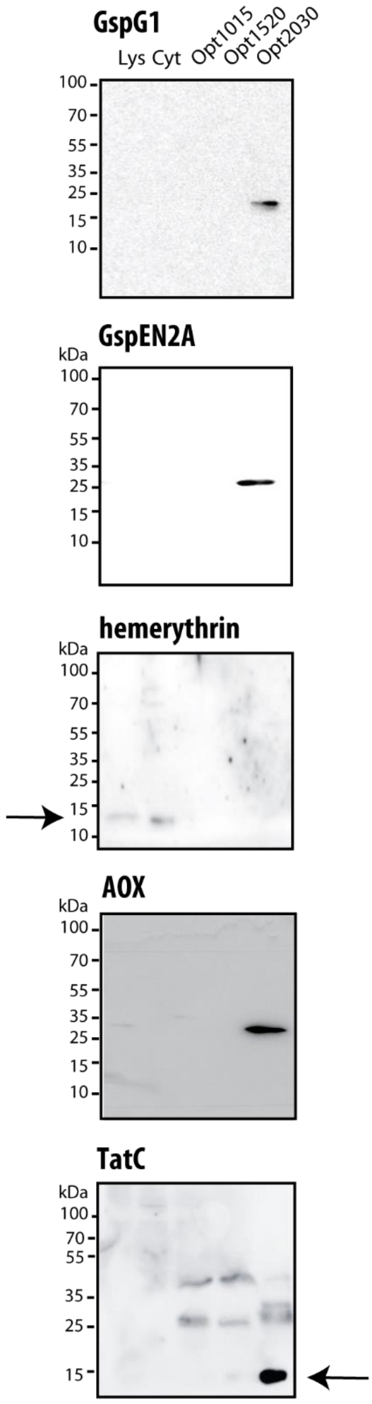

B

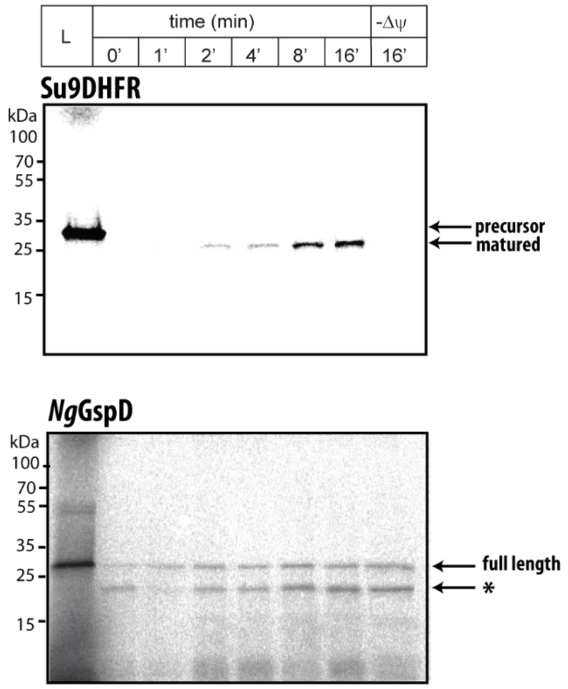

Supplementary Fig. 6. A. Example of western blot analysis using specific polyclonal antibodies raised against GspG1, GspEN2A, hemerythrin and TatC of *N. gruberi* and human alternative oxidase AOX*.* L -lysate, C – cytosol, Opt1015, Opt1520, Opt2030 – sub-fractions of high-speed pellet fraction obtained by gradient centrifugation. **B.** the protein import assays of synthetic Su9DHFR construct and *N. gruberi* GspD into isolated yeast mitochondria, L- loading control, the asterisk denotes cleavage product of *Ng*GspD by trypsin shaving (representative image are shown)

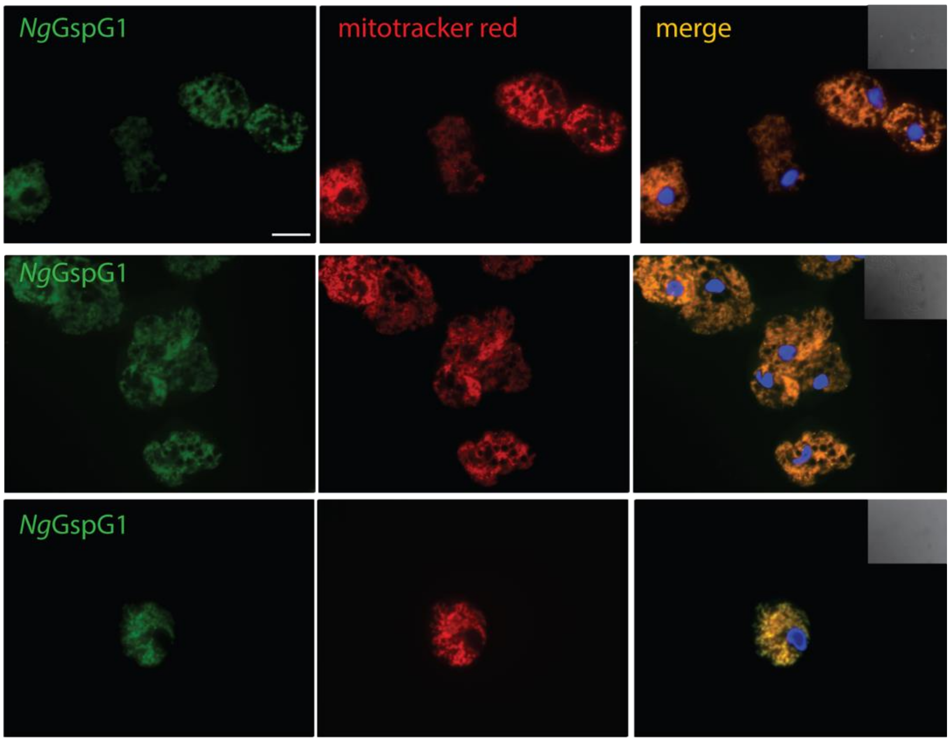

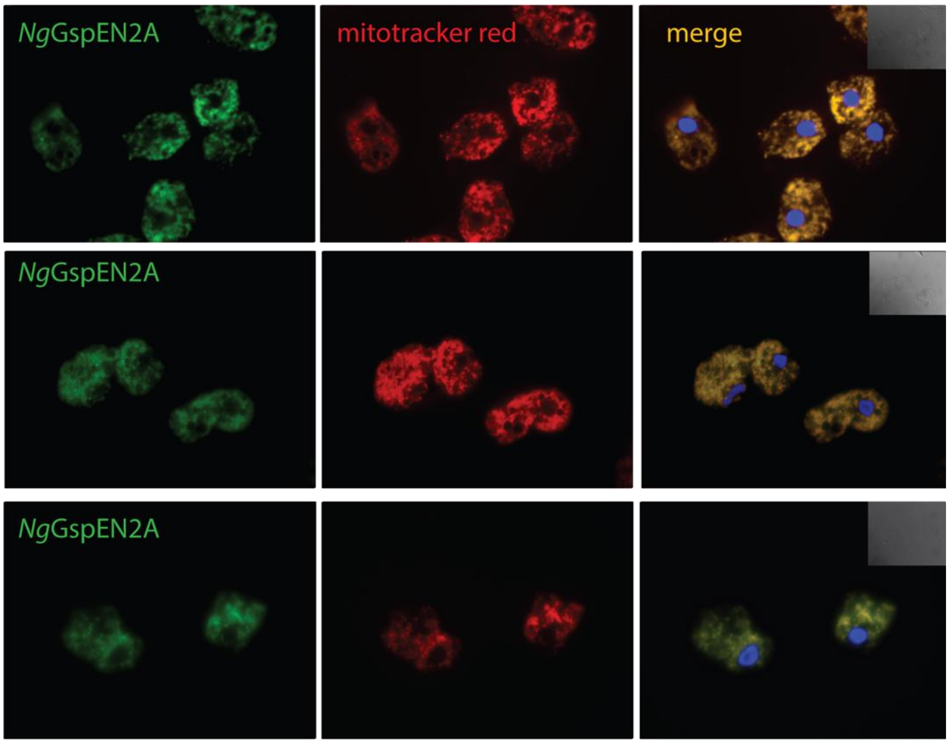

Supplementary Fig. 7. Additional images of immunofluorescence microscopy detection of *Ng*GspG1 and *Ng*GspEL2. Scale bar 10μm. (representative images of three experiments are shown)

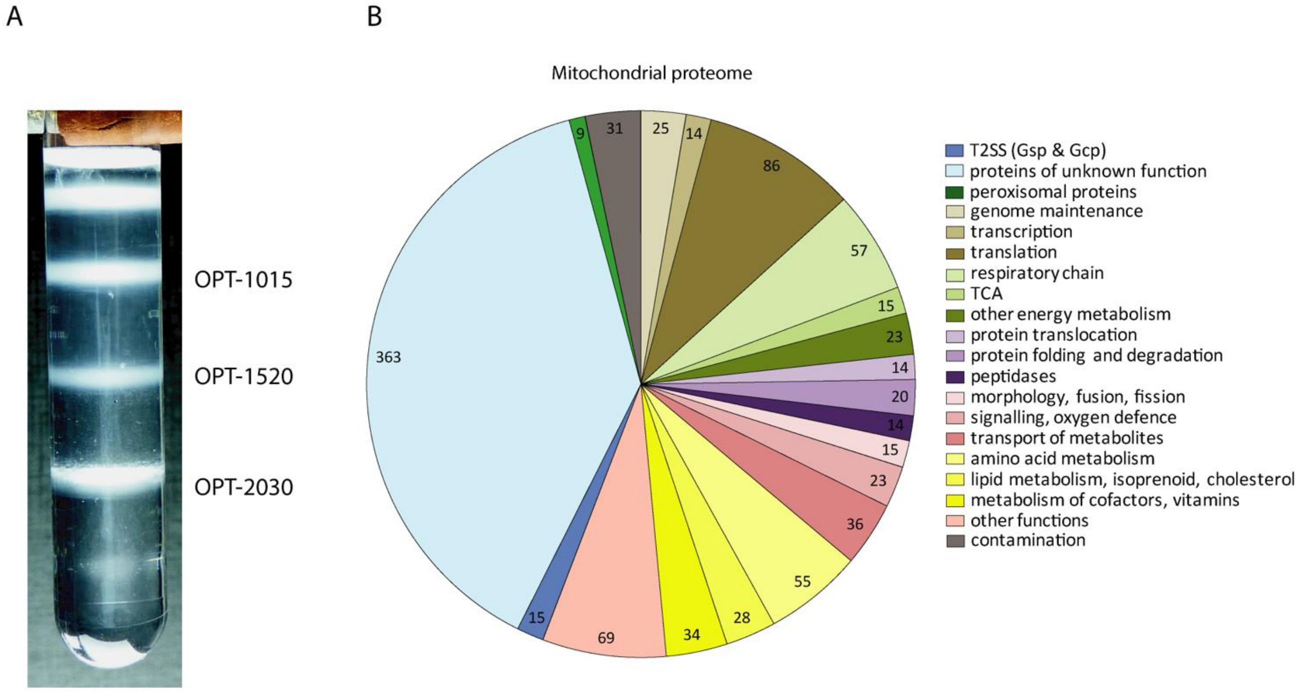

Supplementary Fig. 8 Functional annotation of the putative mitochondrial proteome of *N. gruberi*. **A.** *N. gruberi* cell lysate was further separated into three fractions on Optiprep gradient and proteins extracted from these fractions were then digested with trypsin and peptides were separated by nanoflow liquid chromatography and analyzed by tandem mass spectrometry. **B.** In total 946 putative mitochondrial proteins were identified, which were sorted into functional categories using BLAST and HHpred. The number of proteins in each category is indicated within the respective segment of the pie chart.

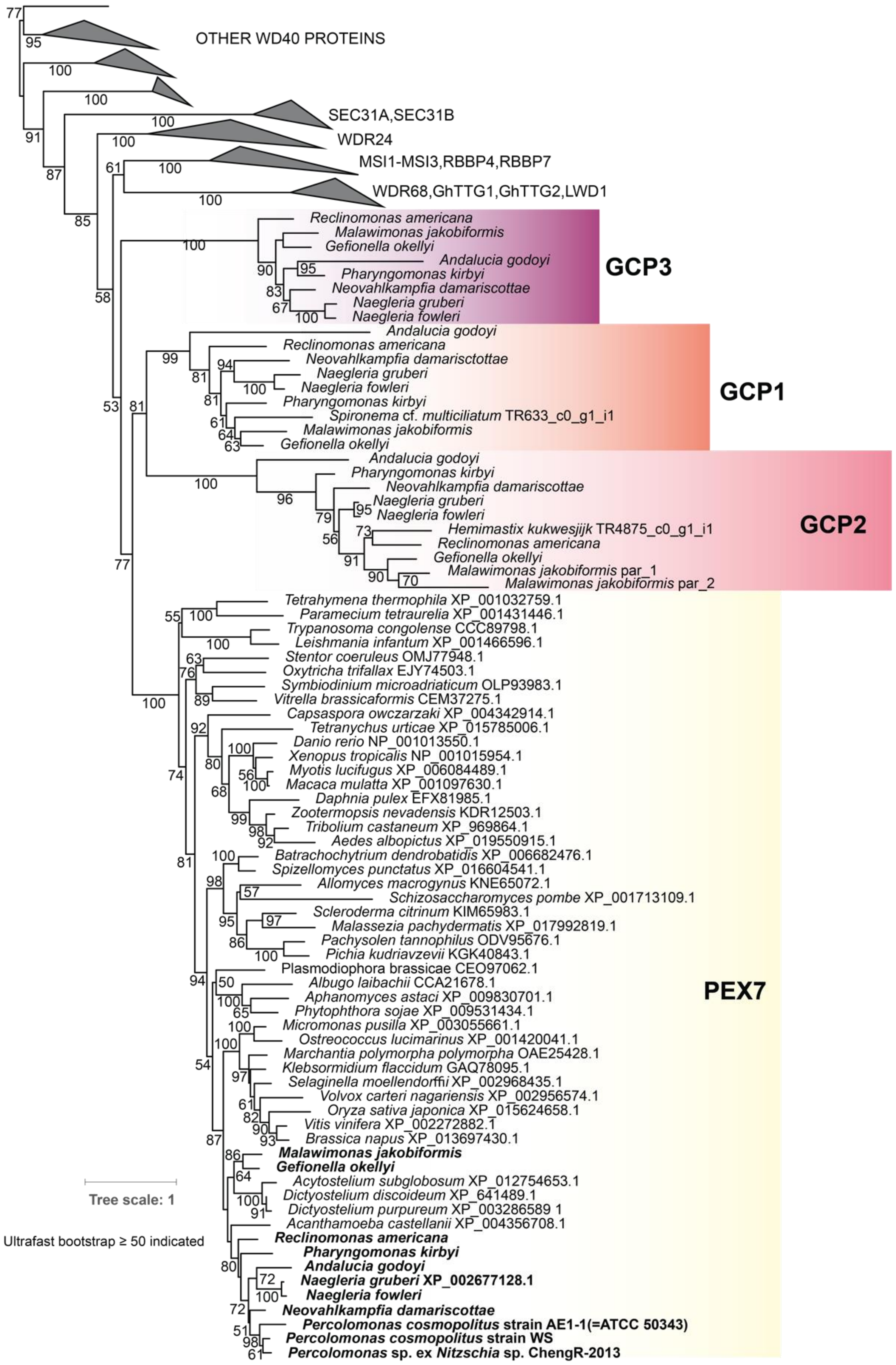

Supplementary Fig. 9 Phylogenetic analysis of the WD40 superfamily including the novel members Gcp1, Gcp2, and Gcp3. The ML tree (IQ-TREE, LG+C60+G, 1000 ultrafast bootstraps, bnni) demonstrates the monophyly of each novel paralogue and suggests that they are specifically related to the peroxisome import protein Pex7. Note that the species possessing Gcp1 to Gcp3 contain also Pex7 itself (in bold).

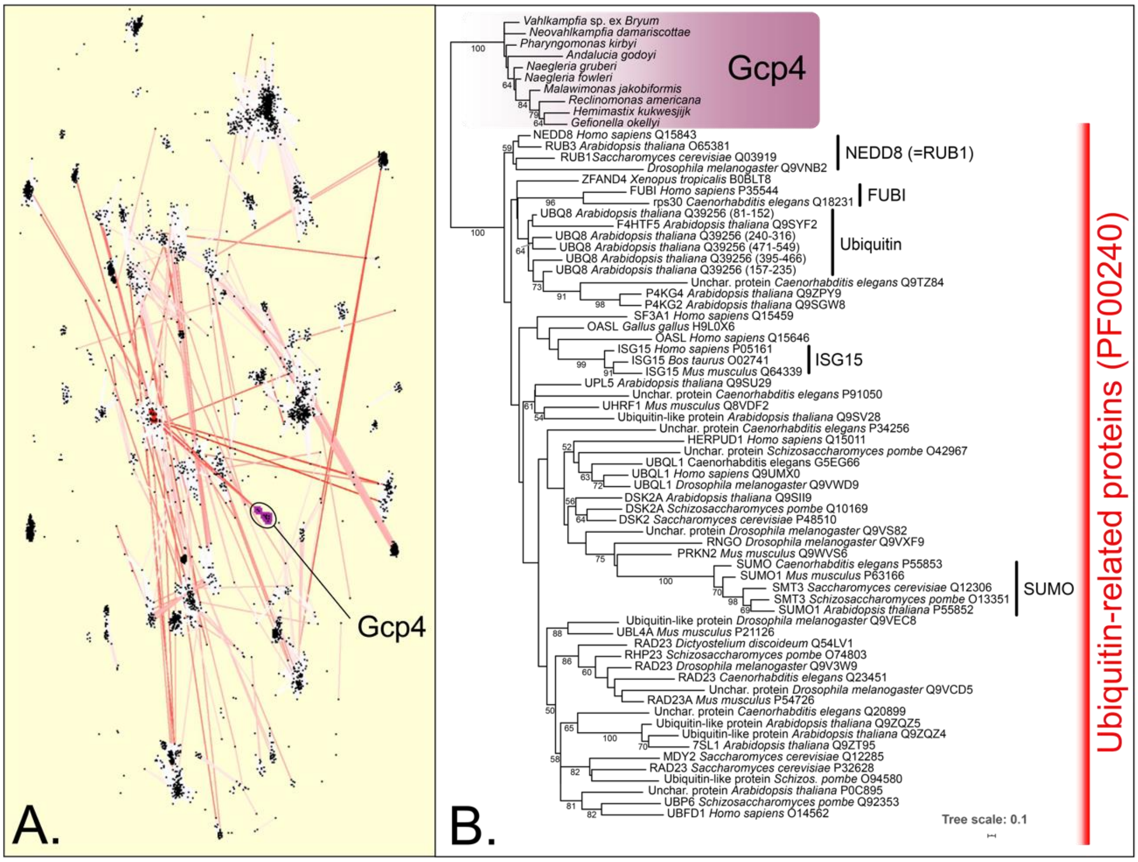

Supplementary Fig. 10 Gcp4 proteins and their relationship to other members of the clan Ubiquitin (CL0072) as defined in Pfam v.31 database (https://pfam.xfam.org). **A.** Cluster analysis of ubiquitin fold of 4764 representative sequences (seed alignments) of the clan Ubiquitin containing members of all 59 defined families. This analysis was computed using the cluster analysis implemented in the CLANS (CLuster ANalysis of Sequences) program with following parameters: BLOSUM62 scoring matrix; extract BLAST HSPs up to E-value of 1e-4; 10,000 rounds. The analysis shows that Gcp4 sequences constitute a novel separate group with a weak affinity to the cloud containing proteins from ubiquitin family (PF00240) and Rad60 SUMO-like family (PF11976). Gcp4 proteins are marked in purple, four proteins directly connected to the Gcp4 cluster and situated in a cloud composed from ubiquitin and Rad60 SUMO-like families are marked in red (R5RDZ3_9PROT, NEDD8_HUMAN, RUB3_ARATH, A8BJ08_GIAIC). Connections between sequences are coloured by edge “frustration” (red=too long; blue=too short). **B.** Phylogenetic analysis of Gcp4 and ubiquitin-related proteins. Gcp4 sequences were added to the seed alignment of the ubiquitin domain as defined in the Pfam v.31 database (PF00240) and the ML tree was calculated using IQ-TREE multicore version 1.5.5 under the LG+G4 model with 10,000 ultrafast bootstraps. The tree is arbitrarily rooted between Gcp4 proteins and the remaining sequences included in the analysis. Sequences assigned to named clades correspond to different type I ubiquitin-like proteins (i.e. those that are conjugated to substrate proteins), the remaining sequences correspond to Type II ubiquitin-like proteins (i.e., larger proteins including a ubiquitin-like domain within a more complex domain architecture.

Gcp5

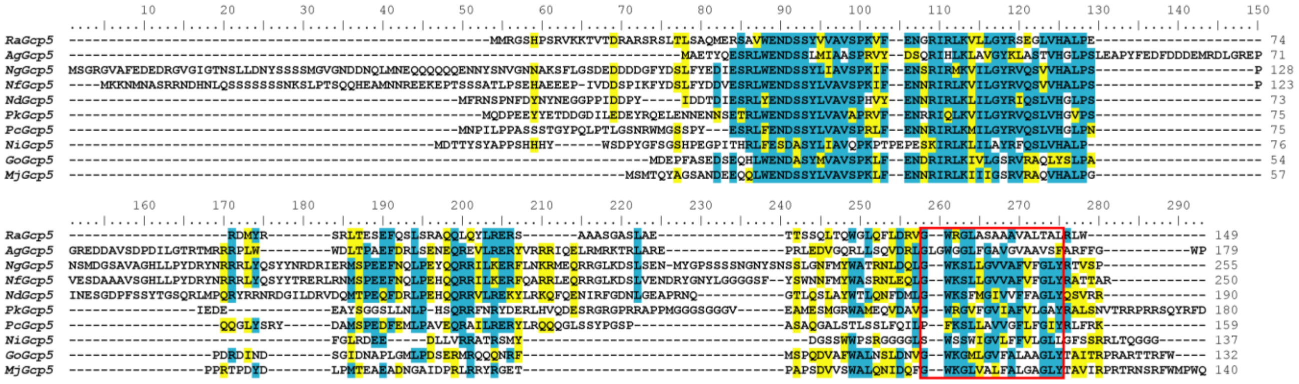

Gcp7

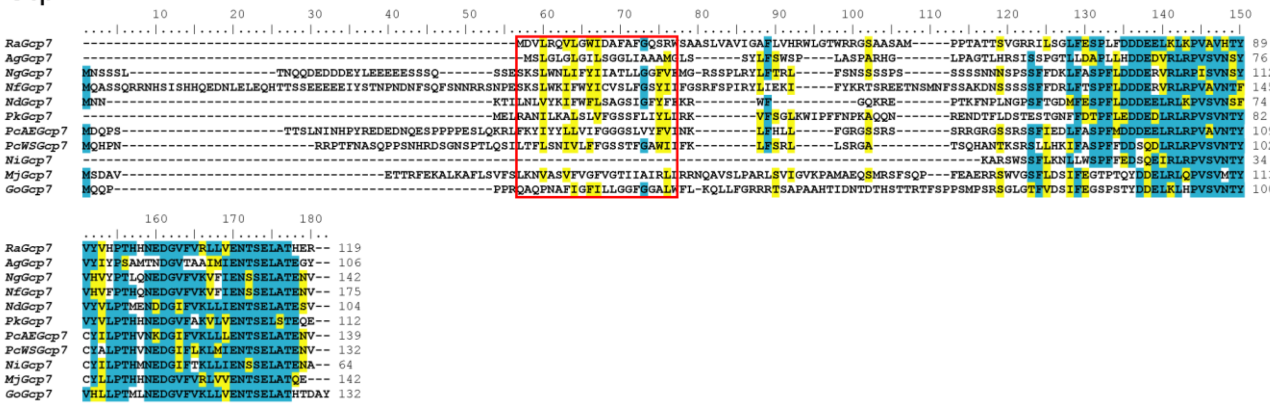

Gcp11

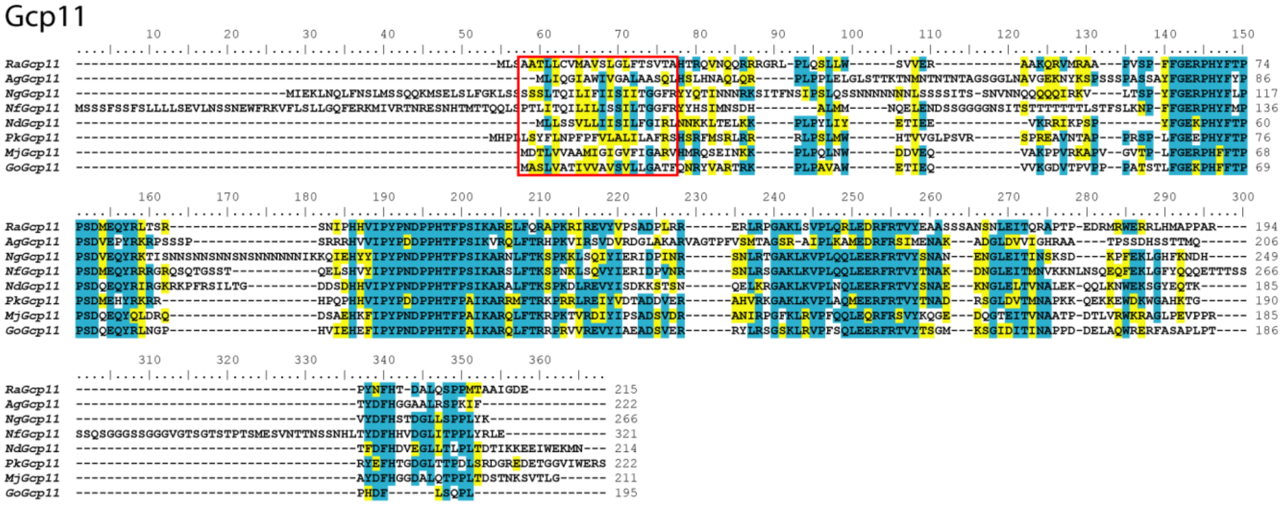

Supplementary Fig. 11 (pg. 30 and 31) Multiple sequence alignments of Gcp5, Gcp7, Gcp11, and Gcp15 proteins. Regions including predicted transmembrane domains are framed by red rectangles. Identical and similar residues are highlighted in turquoise and yellow, respectively (using 50% conservation of the position as the threshold). Species abbreviations: *Mj – Malawimonas jakobiformis*, *Go – Gefionella okellyi*, *Ra – Reclinomonas americana*, *Ag – Andalucia godoyi*, *Nd – Neovahlkampfia damariscottae*, *Pk – Pharyngomonas kirbyi*, *Ng – Naegleria gruberi*, *Nf – Naegleria fowleri*, *PcWS – Percolomonas cosmopolitus* strain WS, *PcAE – Percolomonas cosmopolitus* strain AE-1, *Ni – Percolomonas* sp. contaminating a transcriptome assembly from *Nitzschia* sp. ChengR-2013. *Trit – Naegleria* sp. contaminating a transcriptome assembly from *Triticum polonicum*

Gcp15

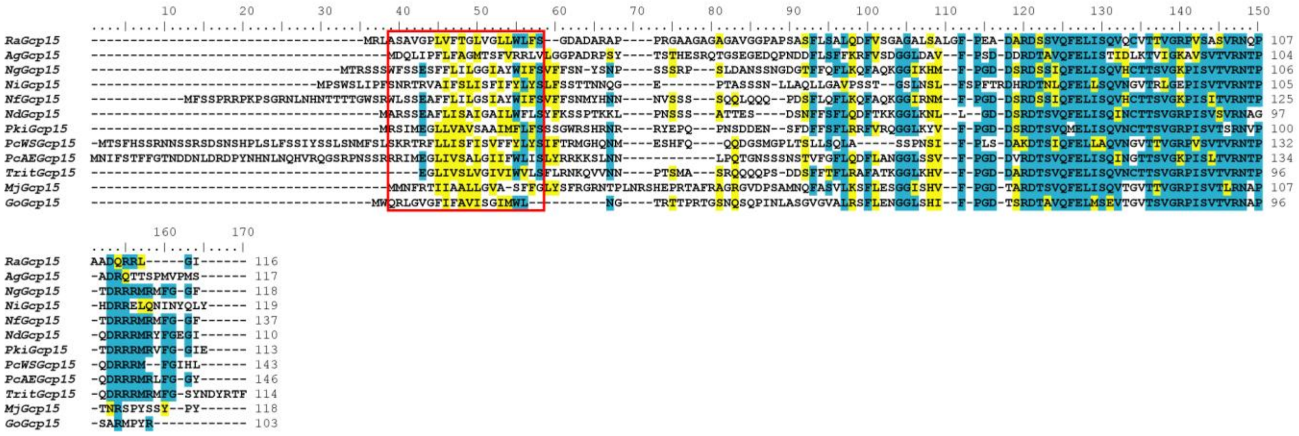

Supplementary Fig. 11 cont. For further details see page 30.

Gcp6

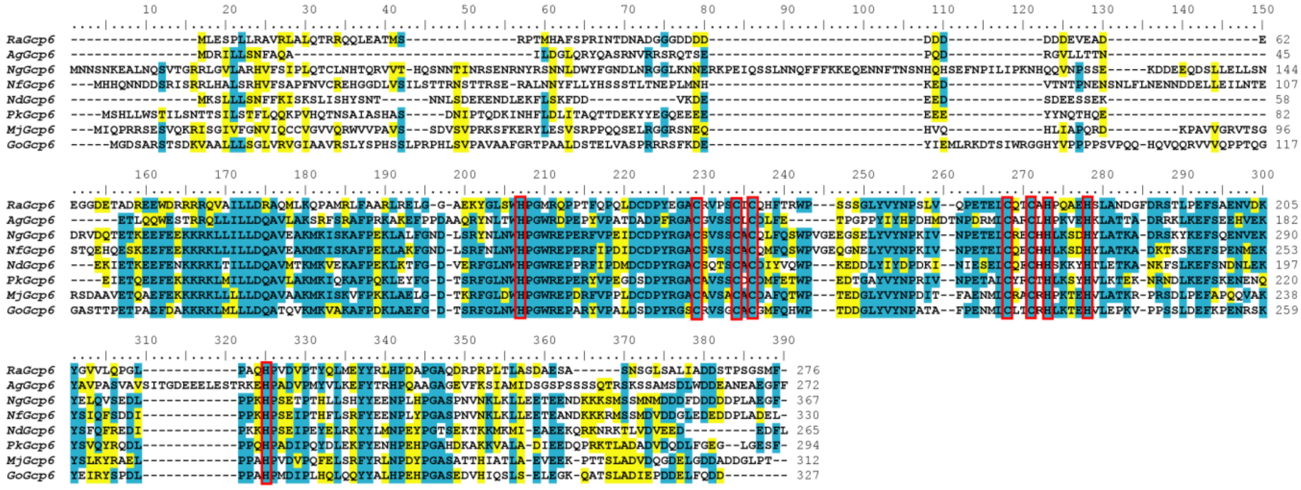

Gcp12

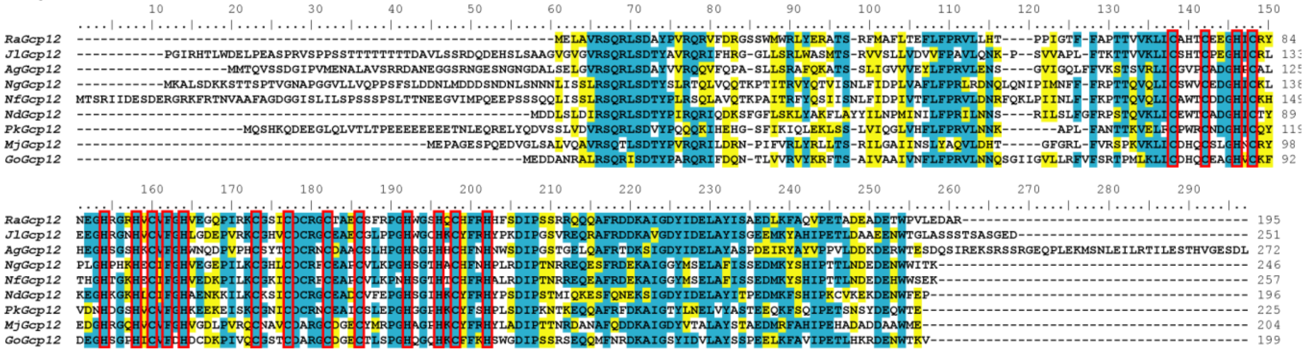

Supplementary Fig. 12 Multiple sequence alignments of Gcp6 and Gcp12 proteins. Absolutely conserved histidine and cysteine residues (indicative of possible binding of a prosthetic group by the proteins) are framed by red rectangles. Identical and similar residues are highlighted in turquoise and yellow, respectively (using 50% conservation of the position as the threshold). Species abbreviations: *Mj – Malawimonas jakobiformis*, *Go – Gefionella okellyi*, *Ra – Reclinomonas americana*, *Ag – Andalucia godoyi*, *Nd – Neovahlkampfia damariscottae*, *Pk – Pharyngomonas kirbyi*, *Ng – Naegleria gruberi*, *Nf – Naegleria fowleri*, *Jl –* Jakoba libera.

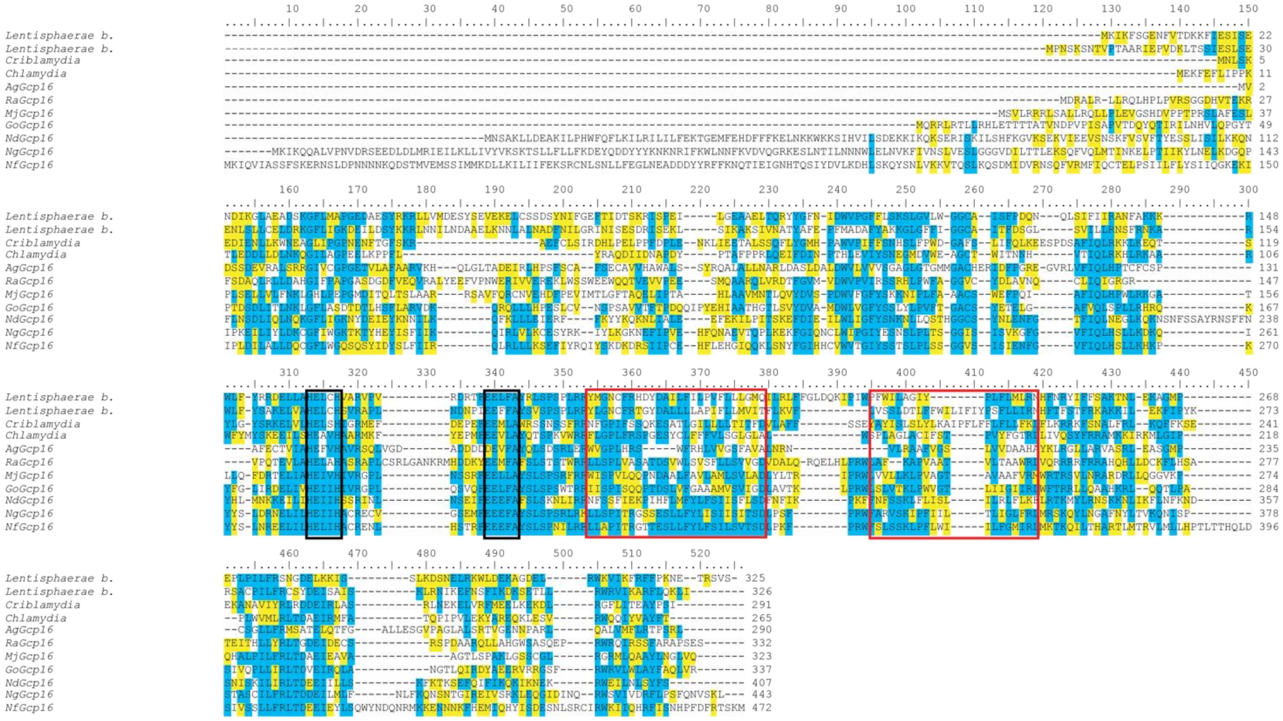

Supplementary Fig. 13 Multiple sequence alignment of the eukaryotic Gcp16 proteins and selected bacterial homologues. Regions including predicted transmembrane domains are framed by red rectangles. Metallopeptidase motifs <u>HEXXH</u> and <u>EXXA</u> are highlighted by the black rectangle. Putative mitochondrial targeting presequences predicted by MitoFates are underlined. Identical and similar residues are highlighted in turquoise and yellow, respectively (using 50% conservation of the position as the threshold). Species abbreviations: *Mj – Malawimonas jakobiformis*, *Go – Gefionella okellyi*, *Ra – Reclinomonas americana*, *Ag – Andalucia godoyi*, *Nd – Neovahlkampfia damariscottae*, *Pk – Pharyngomonas kirbyi*, *Ng – Naegleria gruberi*, *Nf – Naegleria fowleri*.

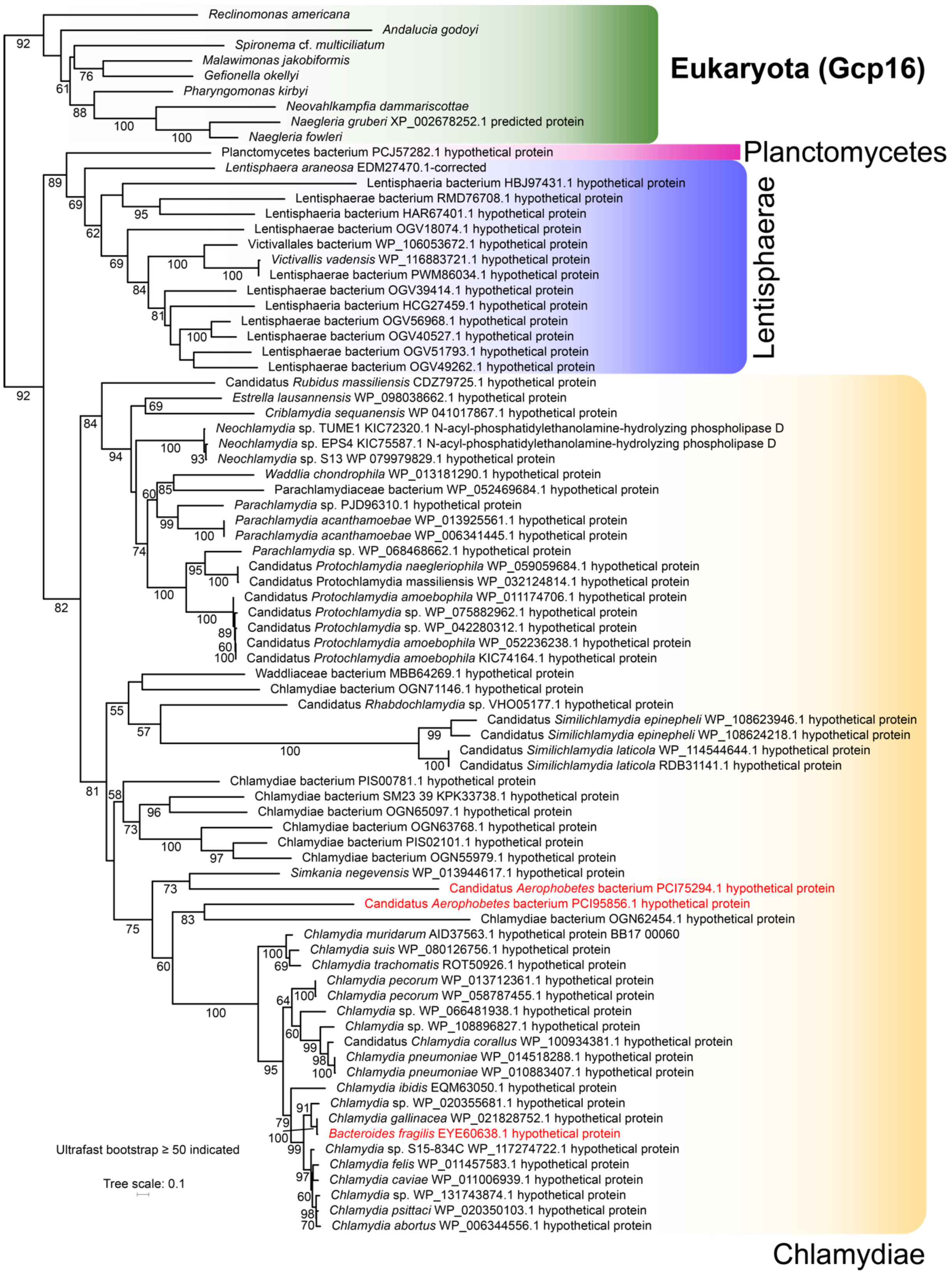

Supplementary Fig. 14 Phylogenetic analysis of the Gcp16 protein. The ML tree (IQ-TREE, substitution model LG+R4, 1000 ultrafast bootstraps) includes all Gcp16 homologues identified in databases (using an exhaustive psi-blast search of the NCBI nr protein database and targeted blast searches of genomic and transcriptomic data from other repositories). The three sequences highlighted in red come from apparently misidentified taxa or contaminated genome assemblies. The sequence EYE60638.1 is encoded by a gene on a >800 kbp contig attributed to *Bacteroides fragilis* str. S6L5, i.e. a member of the phylum Bacteroidetes, but blast searches with proteins encoded by randomly picked genes from different

regions of the contig all give as identical hits sequences from *Chlamydia gallinacea*. The two sequences attributed to two different isolates of an "Candidatus Aerophobetes bacterium" (i.e. members of the phylum Candidatus Aerophobetes) are located on contigs encoding proteins that exclusively (in case of PCI75294.1) or predominantly (PCI95856.1) retrieve sequences from the phylum Chlamydiae as best non-self hits, hence are most likely derived from unidentified representatives of the latter phylum.

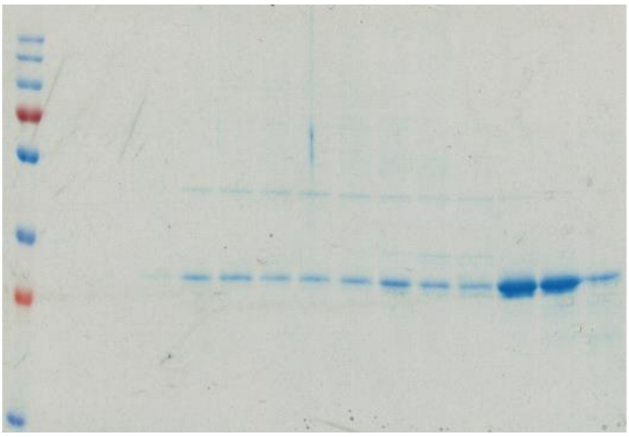

Supplementary Figure 15. Full scan of size exclusion chromatography of *Ng*GspD

Supplementary Methods

Sequencing and assembly of *Neovahlkampfia damariscottae* and "*Malawimonas californiana*" genomes

Neovahlkampfia damariscottae CCAP 1588/7 (obtained from the Culture Collection of Algae and Protozoa; https://www.ccap.ac.uk/) was grown for 5 days in 20 Corning® cell culture flasks (surface area 25 cm2) with 10 ml of ATCC 1525 medium. Since the culture contained various bacteria, we incubated cells overnight in 10 ml of fresh ATCC 1525 medium with 166 µl of antibiotics prepared as a mixture of ampicillin (100 µl), streptomycin (500 µl), penicillin (250 µl), and kanamycin (50 µl). Genome DNA was extracted using Qiagen DNeasy Blood & Tissue Kit following the manufacturer´s instructions. Genomic DNA was sequenced on Illumina Miseq 2x250bp platform. The total of 27,972,096 reads were trimmed by Trimmomatic [3]. An initial assembly was built with Spades 3.10.1 [4] and the scaffolds were clustered into different bins by MaxBin [5]. Because the average GC content of the *N. damariscottae* genome is low (27.4% in the final assembly), bins with a relatively high GC content (> 40%) were inspected manually and after confirmation of their bacterial origin, they were removed from the assembly. Trimmed reads were mapped back to the cleaned assembly with Bowtie2 (--very-sensitive-local settings; [6] and 14,663,323 mapped reads were used for the final assembly, which was built with Spades 3.10.1 using the "careful" option. The final assembly consists of 1,865 scaffolds with the cumulative length of 21.5 Mb, and the N50 value of 95,418 bp. It was deposited at GenBank with the accession number JABLTG000000000.

"*Malawimonas californiana*" (ATTC 50740) is a formally undescribed malawimonad that is cultivated with live *Enterobacter aerogenes* (ATCC 13048) as a food source (detailed recipes for the media are described at http://megasun.bch.umontreal.ca/People/lang/FMGP/methods.html). The large variety of food bacteria that was present in the original strain was reduced by repeated dilutions in 100 mL growth medium plus live *E. aerogenes*, so as to retain only a few malawimonad cells, and grown to the early stationary phase. The final isolate with less bacterial contaminants (after five growth cycles) is being kept for long-term storage under liquid nitrogen. For DNA purification, aliquots of the stock culture were added to fresh medium (500 mL, 2.5 L Erlenmeyer flasks) containing ~200 mg pre-cultured live *E. aerogenes* cells. Cultures were gently shaken at 22°C and daily supplemented with live bacteria, provided that most bacterial cells were consumed. Cells were harvested by centrifugation in the early stationary growth phase (after 2-5 days), at a point when most food bacteria were consumed. Harvested cells were lysed in a Tris-EDTA buffer containing 0.2 % SDS plus 100 µg/ml proteinase K, dialyzed for 24 hr against the same buffer, and then further purified by CsCl-*bis*benzimide equilibrium gradient centrifugation [7]. GS FLX Library Preparation Method protocols (Roche) were used to prepare shotgun and paired-end (3 Kb and 8 Kb) libraries for sequencing with the 454 method on the Titanium platform. Altogether, 2,955,688 shotgun reads, 1,082,625 reads from the 3 Kb paired-end libraries, and 1,100,119 reads from the 8 Kb

paired-end libraries were generated and assembled using Newbler 2.8 (Roche), yielding a draft genome assembly consisting of 1,123 scaffolds with the cumulative length of 51.4 Mb, and the N50 value of 399,050 bp. The assembly was annotated by using Augustus [8], yielding 13,534 predicted protein sequences. The assembly and the predicted protein sequence set are available at http://megasun.bch.umontreal.ca/Malawimonas_californiana. Note that the assembly is not bacterial contamination-free and is expected to be to a certain degree inaccurate in homopolymeric regions, owing to the known limitation of the 454 sequencing method. Work on a more accurate assembly and annotation is underway and the outcomes will be published elsewhere.

Sequence curation

Existing Gsp and Gcp gene models (public for the *N. gruberi* genome and private for *A. godoyi* and the two malawimonads) were evaluated by considering transcriptome data and sequence conservation within the respective gene families, and modified if necessary. One of the *N. gruberi* genes proved to be affected by an assembly error in the existing genome assembly; this was corrected by consulting the original Sanger sequencing reads. For genes without existing gene models (i.e. genes missed by automated annotation programs or residing in as-yet unannotated genome assemblies), the respective gene models were built manually *de novo* using the same source of supporting information. For *R. americana* only three alternative fragmented genome assemblies, often with redundant highly similar contigs presumably representing alternative allele variants, were available; hence, transcriptome data were used to link separate contigs containing different parts (exons) of the genes. The resulting gene assemblies may thus be chimeras of different alleles.

Only transcriptomic data were available for *P. kirbyi* and the three *Percolomonas* spp., so the Gsp and Gcp protein sequences were deduced by conceptual translation from the appropriate reading frames of the relevant transcript contigs. In several cases the original contigs from transcriptome assemblies proved to include a truncated coding sequence (CDS); in most of these cases a complete CDS could be obtained by manual iterative extension of the truncated ends by using raw Illumina reads identified by blastn searches and considering linking information provided by paired-end reads. Note that for one of the *Percolomonas* strains, the transcriptomic data are available only as contamination in the transcriptome assembly attributed to the diatom *Nitzschia* sp. ChengR-2013 (GenBank accession number GBCF00000000.1). The taxonomic assignment of the respective Gsp/Gcp sequences to *Percolomonas* rather than *Nitzschia* is based on the presence in the assembly of a 18S rRNA sequence phylogenetically close to a sequence from *Percolomonas cosmopolitus* strain WS (Supplementary fig. 1) and considering the fact that homologues are absent from all other diatom (even stramenopile) transcriptomic and genomic assemblies.

In case of Gcp4 a homologue was found in a transcriptome assembly attributed to the moss *Bryum argenteum* (GenBank accession number GCZP00000000.1), i.e. an organism lacking other Gsp and Gcp homologues. The sequence is most likely a contaminant coming from a heterolobosean, specifically a V*ahlkampfia* sp., based on the presence in the assembly of a partial 18S rRNA sequence (accession number GCZP01036684.1) exhibiting 99% identity to the 18S rRNA sequence from *Vahlkampfia avara* strain 4171L. It is likely that *Vahlkampfia*, like many other heteroloboseans, exhibits a full set of Gsp/Gcp homologues, which are not represented in the assembly GCZP00000000.1 due to an insufficient coverage of the contaminating organism.

Defining orthogroups for phylogenetic profiling

In order to classify eukaryotic genes into putative groups of orthologous genes (orthogroups) for the purpose of identification of genes co-occurring with initially identified Gsp homologues in eukaryotes (phylogenetic profiling), two slightly different rounds analyses were performed. They differed in taxon sampling (Supplementary Table 6; the first round lacking protein sequences from *G. okellyi* and *Monocercomonoides exilis*), sequence similarity thresholds (see step p1 below) and the way how week connections between sequence clusters were dealt with (see step n3 below).
The analysis included two main phases. In the first, orthologous relationships of each protein to all others were inferred using the following custom bioinformatics pipeline (steps p1 to p4 were repeated for each protein):
_ (p1) the protein was blasted (BlastP) against the entire set of proteins using a threshold e-value ≤ 1e-8 and the BlastP alignment overlapping at least 35 % of the query (first analysis) or minimal e-value threshold of 1e-5 and a the BlastP alignment overlapping at least 25 % of the query (second analysis).
_ (p2) the seven best BlastP hits of each proteome were retrieved, aligned with Clustal Omega [9] under default parameters, and the resulting alignment was trimmed using trimAl [10] by removing columns with gaps in more than 50% of sequences.
_ (p3) a phylogenetic tree was built from the trimmed alignment using FastTree [11] under default parameters.

_ (p4) orthologous relationships were inferred from the mid-point rooted phylogenetic tree using the specie-overlap method implemented in ETE [12].

All orthologous relationships obtained from these phylogenetic analyses were combined to build an undirected graph in which vertices represent proteins, edges represent observed orthologous relationships between the two connected proteins, and connected components represent orthologous groups. This complex and dense network was then simplified using these successive steps:

_ (n1) edges connecting two proteins that have been found orthologous only once were discarded.

_ (n2) for each edge, we calculated the ratio “number of times the two proteins have been found orthologous) / (number of times the two proteins have been found paralogous”. Edges with the ratio lower than 0.5 were discarded.

_ (n3) bridges, identified as edges connecting two vertices that have at least four neighbours and that do not share a connected vertex, were discarded. This step was applied only in the second analysis.

_ (n4) within each orthologous group, gene-fusions were identified and the orthologous group was corrected as follows: if the removal of a vertex led to the formation of two isolated connected components of at least three vertices, the vertex was considered as a gene fusion (genuine or artificial) and was added to each of the two newly formed connected components. To validate the approach, we then inspected 30 randomly chosen orthologous groups (including some belonging to complex gene families) and found rare false positive and false negative cases. Additionally, 20 randomly chosen proteins identified by our pipeline as gene fusions (an average of 31 gene fusions were inferred per proteome) were all confirmed by manual BlastP analyses.

Supplementary References

1. Abby, S. S. *et al.* Identification of protein secretion systems in bacterial genomes. *Sci. Rep.* **6**, 23080 (2016).
2. Fukasawa, Y. *et al.* MitoFates: improved prediction of mitochondrial targeting sequences and their cleavage sites. *Mol. Cell. Proteomics* **14**, 1113–26 (2015).
3. Bolger, A. M., Lohse, M. & Usadel, B. Trimmomatic: A flexible trimmer for Illumina sequence data. *Bioinformatics* **30**, 2114–2120 (2014).
4. Bankevich, A. *et al.* SPAdes: A new genome assembly algorithm and its applications to single-cell sequencing. *J. Comput. Biol.* **19**, 455–477 (2012).
5. Wu, Y. W., Tang, Y. H., Tringe, S. G., Simmons, B. A. & Singer, S. W. MaxBin: An automated binning method to recover individual genomes from metagenomes using an expectation-maximization algorithm. *Microbiome* **2**, (2014).
6. Langmead, B. & Salzberg, S. L. Fast gapped-read alignment with Bowtie 2. *Nat. Methods* **9**, 357–359 (2012).
7. Lang, B. F. & Burger, G. Purification of mitochondrial and plastid DNA. *Nat. Protoc.* **2**, 652–660 (2007).
8. Hoff, K. J. & Stanke, M. WebAUGUSTUS--a web service for training AUGUSTUS and predicting genes in eukaryotes. *Nucleic Acids Res.* **41**, (2013).
9. Sievers, F. *et al.* Fast, scalable generation of high-quality protein multiple sequence alignments using Clustal Omega. *Mol. Syst. Biol.* **7**, 539 (2011).
10. Capella-Gutiérrez, S., Silla-Martínez, J. M. & Gabaldón, T. trimAl: A tool for automated alignment trimming in large-scale phylogenetic analyses. *Bioinformatics* **25**, 1972–1973 (2009).
11. Price, M. N., Dehal, P. S. & Arkin, A. P. FastTree: computing large minimum evolution trees with profiles instead of a distance matrix. *Mol. Biol. Evol.* **26**, 1641–50 (2009).
12. Huerta-Cepas, J., Dopazo, J. & Gabaldón, T. ETE: a python Environment for Tree Exploration. *BMC Bioinformatics* **11**, 24 (2010).
