# Supplementary material for: Analysis of diverse eukaryotes suggests the existence of an ancestral mitochondrial apparatus derived from the bacterial type II secretion system
Source: Nat Commun. 2021 May 19;12:2947. doi: 10.1038/s41467-021-23046-7 (PMC8134430; doi:10.1038/s41467-021-23046-7)
Supplement: Supplementary file 2 — Description of Additional Supplementary Files [file 41467_2021_23046_MOESM2_ESM.pdf]

Description of Additional Supplementary Files

Supplementary Data 1. Gsp and Gcp protein sequences from the eukaryotic species specifically targeted in the study and their features.

Supplementary Data 2. Mitochondrial and peroxisomal proteome of *N. gruberi*.

Supplementary Data 3. Gsp and Gcp sequences from non-target organisms.

Supplementary Data 4. Sequence data sources.

Supplementary Data 5. Species and their predicted proteomes used for the initial step of the search for Gsp-co-occurring genes in eukaryotes.

Supplementary Data 6. PCR primers used in the study.
