# Supplementary material for: Analysis of diverse eukaryotes suggests the existence of an ancestral mitochondrial apparatus derived from the bacterial type II secretion system
Source: Nat Commun. 2021 May 19;12:2947. doi: 10.1038/s41467-021-23046-7 (PMC8134430; doi:10.1038/s41467-021-23046-7)
Supplement: Supplementary file 10 — Reporting Summary [file 41467_2021_23046_MOESM10_ESM.pdf]

nature research

Corresponding author(s): Pavel Dolezal, Marek Elias

Last updated by author(s): Mar 6, 2021

Reporting Summary

Nature Research wishes to improve the reproducibility of the work that we publish. This form provides structure for consistency and transparency in reporting. For further information on Nature Research policies, see our Editorial Policies and the Editorial Policy Checklist.

Statistics

For all statistical analyses, confirm that the following items are present in the figure legend, table legend, main text, or Methods section.

n/a	Confirmed	
[ ]	[x]	The exact sample size (*n*) for each experimental group/condition, given as a discrete number and unit of measurement
[x]	[ ]	A statement on whether measurements were taken from distinct samples or whether the same sample was measured repeatedly
[x]	[ ]	The statistical test(s) used AND whether they are one- or two-sided<br>*Only common tests should be described solely by name; describe more complex techniques in the Methods section.*
[x]	[ ]	A description of all covariates tested
[x]	[ ]	A description of any assumptions or corrections, such as tests of normality and adjustment for multiple comparisons
[ ]	[x]	A full description of the statistical parameters including central tendency (e.g. means) or other basic estimates (e.g. regression coefficient) AND variation (e.g. standard deviation) or associated estimates of uncertainty (e.g. confidence intervals)
[x]	[ ]	For null hypothesis testing, the test statistic (e.g. *F*, *t*, *r*) with confidence intervals, effect sizes, degrees of freedom and *P* value noted<br>*Give P values as exact values whenever suitable.*
[x]	[ ]	For Bayesian analysis, information on the choice of priors and Markov chain Monte Carlo settings
[x]	[ ]	For hierarchical and complex designs, identification of the appropriate level for tests and full reporting of outcomes
[x]	[ ]	Estimates of effect sizes (e.g. Cohen's *d*, Pearson's *r*), indicating how they were calculated

Our web collection on statistics for biologists contains articles on many of the points above.

Software and code

Policy information about availability of computer code

Data collection	no software was used
Data analysis	HHpred (https://toolkit.tuebingen.mpg.de/#/tools/hhpred), Phyre2 server(http://www.sbg.bio.ic.ac.uk/phyre2/html/page.cgi?id=index), MUSCLE - Edgar, R. C. MUSCLE: multiple sequence alignment with high accuracy and high throughput. Nucleic Acids Res. 32, 1792–7 (2004), HMMER - Finn, R. D., Clements, J. & Eddy, S. R. HMMER web server: interactive sequence similarity searching. Nucleic Acids Res. 39, W29-37 (2011), BMGE - Criscuolo, A. & Gribaldo, S. BMGE (Block Mapping and Gathering with Entropy): a new software for selection of phylogenetic informative regions from multiple sequence alignments. BMC Evol. Biol. 10, 210 (2010), IQ-TREE- Nguyen, L.-T., Schmidt, H. A., von Haeseler, A. & Minh, B. Q. IQ-TREE: a fast and effective stochastic algorithm for estimating maximum-likelihood phylogenies. Mol. Biol. Evol. 32, 268–74 (2015), SWISS-MODEL server - Bienert, S. et al. The SWISS-MODEL Repository-new features and functionality. Nucleic Acids Res. 45, D313–D319 (2017), Guex, N., Peitsch, M. C. & Schwede, T. Automated comparative protein structure modeling with SWISS-MODEL and Swiss-PdbViewer: A historical perspective. Electrophoresis 30, S162–S173 (2009). Biasini, M. et al. SWISS-MODEL: modelling protein tertiary and quaternary structure using evolutionary information. Nucleic Acids Res. 42, W252-8 (2014), ImageJ 1.52p, SVI Huygens Professional software 19.10, LAS-X_3.5.6.21594.

For manuscripts utilizing custom algorithms or software that are central to the research but not yet described in published literature, software must be made available to editors and reviewers. We strongly encourage code deposition in a community repository (e.g. GitHub). See the Nature Research guidelines for submitting code & software for further information.

Data

Policy information about availability of data

All manuscripts must include a data availability statement. This statement should provide the following information, where applicable:

- Accession codes, unique identifiers, or web links for publicly available datasets
- A list of figures that have associated raw data
- A description of any restrictions on data availability

All sequences of Gsp and Gcp proteins analysed in the study are provided in Supplementary Table 1. Gsp and Gcp genes extracted from an unpublished Malawimonas jakobiformis genome assembly have been deposited at GenBank with accession numbers MT460910-MT460938. Raw genome sequencing reads from "Malawimonas californiana" Neovahlkampfia damariscottae are available from NCBI under the BioProject PRJNA549687. Genome assembly from N. damariscottae has been deposited at GenBank with accession number JABLTG000000000. Transcriptome assembly of Gefionella okellyi, genome assembly and predicted proteins from "Malawimonas californiana", partial genome assemblies from Reclinomonas americana are available from https://megasun.bch.umontreal.ca/papers/T2SS-2020/. The mass spectrometry proteomics data have been deposited in the ProteomeXchange Consortium via the PRIDE96 partner repository with the dataset identifier PXD007764, Bryum argenteum transcriptom used (GenBank accession number GCZP00000000.1),
Other relevant data (e.g. multiple sequence alignments used for phylogenetic analyses) are available from the authors upon request.

Field-specific reporting

Please select the one below that is the best fit for your research. If you are not sure, read the appropriate sections before making your selection.

- [x] Life sciences
- [ ] Behavioural & social sciences
- [ ] Ecological, evolutionary & environmental sciences

For a reference copy of the document with all sections, see nature.com/documents/nr-reporting-summary-flat.pdf

Life sciences study design

All studies must disclose on these points even when the disclosure is negative.

Sample size	no sample size calculation was needed, for activity measurements triplicates were performed as usual standard
Data exclusions	no data were excluded
Replication	all attempts of replication were successful, western blots, activity measurements and BN-PAGE were performed at least three times
Randomization	the cells were randomly selected for immunoflurescence microscopy analysis
Blinding	blinding was not performed as other controls were in place

Reporting for specific materials, systems and methods

We require information from authors about some types of materials, experimental systems and methods used in many studies. Here, indicate whether each material, system or method listed is relevant to your study. If you are not sure if a list item applies to your research, read the appropriate section before selecting a response.

Materials & experimental systems

n/a	Involved in the study	
[ ]	[x]	Antibodies
[x]	[ ]	Eukaryotic cell lines
[x]	[ ]	Palaeontology and archaeology
[x]	[ ]	Animals and other organisms
[x]	[ ]	Human research participants
[x]	[ ]	Clinical data
[x]	[ ]	Dual use research of concern

Methods

n/a	Involved in the study	
[x]	[ ]	ChIP-seq
[x]	[ ]	Flow cytometry
[x]	[ ]	MRI-based neuroimaging

Antibodies

Antibodies used	custom made specific polyclonal antibodies produced in rats were raised against GspG1 and GspEN2A, commercial secondary antibodies anti-rat V5 - Abcam - ab206571, anti-rat HRP - Sigma - A9037 and anti-rat Alexa488 - Invitrogen - A-21208 were used.
Validation	custom made antibodies were first verified by the western blot and ELISA using the immnunogenic antigen, secondary antibodies were used according to the manufacturer's protocol.
